# Functional Characterization of microRNA171 Family in Tomato

**DOI:** 10.3390/plants8010010

**Published:** 2019-01-04

**Authors:** Michael Kravchik, Ran Stav, Eduard Belausov, Tzahi Arazi

**Affiliations:** Institute of Plant Sciences, Agricultural Research Organization, Volcani Center, P.O. Box 6, Bet Dagan 50250, Israel; michael.kravchik@mail.huji.ac.il (M.K.); ranstav@volcani.agri.gov.il (R.S.); eddy@volcani.agri.gov.il (E.B.)

**Keywords:** miR171, pollen, STTM, tapetum, callose, tomato

## Abstract

Deeply conserved plant microRNAs (miRNAs) function as pivotal regulators of development. Nevertheless, in the model crop *Solanum lycopersicum* (tomato) several conserved miRNAs are still poorly annotated and knowledge about their functions is lacking. Here, the tomato miR171 family was functionally analyzed. We found that the tomato genome contains at least 11 *SlMIR171* genes that are differentially expressed along tomato development. Downregulation of sly-miR171 in tomato was successfully achieved by transgenic expression of a short tandem target mimic construct (STTM171). Consequently, sly-miR171-targeted mRNAs were upregulated in the silenced plants. Target upregulation was associated with irregular compound leaf development and an increase in the number of axillary branches. A prominent phenotype of *STTM171* expressing plants was their male sterility due to a production of a low number of malformed and nonviable pollen. We showed that sly-miR171 was expressed in anthers along microsporogenesis and significantly silenced upon *STTM171* expression. Sly-miR171-silenced anthers showed delayed tapetum ontogenesis and reduced callose deposition around the tetrads, both of which together or separately can impair pollen development. Collectively, our results show that sly-miR171 is involved in the regulation of anther development as well as shoot branching and compound leaf morphogenesis.

## 1. Introduction

Plant microRNAs (miRNAs) constitute a major class of endogenous small RNAs and trigger the sequence-specific post-transcriptional repression of one to several target mRNAs with high sequence complementarity. The analysis of miRNAs from various land plants species indicated the presence of at least eight deeply conserved miRNA families in all embryophytes [1]. Studies of these miRNAs suggest that most of them act as master regulators of development by negative regulation of the expression of transcription factors that function in critical developmental processes [2,3].

The miR171 family is deeply conserved and exists in all major land plant groups, including bryophytes, one of the oldest groups of land plants [4]. Known plant genomes contain variable number of *MIR171* genes: from only two in *Citrus sinensis* to staggering 21 in *Glycine max* (miRBase release 22). Members of a miR171 family contain one or more nucleotide changes similar to members from other miRNA families, but unlike other conserved miRNAs they may be offset by three nucleotides relative to each other [5]. This atypical sequence offset may result in different target specificities for certain miR171 members [6]. Hitherto, miR171 members have been demonstrated to guide the cleavage of mRNAs coding for GRAS domain SCARECROW-like transcription factors that belong to the HAIRY MERISTEM (HAM) or NODULATION SIGNALING PATHWAY (NSP) clades [6,7,8].

In *Nicotiana benthamiana*, spatial characterization of miR171 expression by in-situ hybridization has shown that it is expressed in a wide variety of tissues including the shoot apical meristem (SAM), leaf primordia, anthers and ovaries, thus hinting on its involvement in their development [9]. In *A. thaliana* (Arabidobsis), ath-miR171b expression was shown to oscillate during the diurnal cycle suggesting a potential role for light in its accumulation [10]. In *Medicago truncatula*, miR171h expression is upregulated in roots during their colonization by arbuscular mycorrhizal fungi or in response to lipochito-oligosaccharides that are released during fungi pre-symbiotic growth, suggesting that miR171h functions to prevent over-colonization of roots by arbuscular mycorrhizal fungi [8]. In rice, it was demonstrated that reduction of osa-miR171b contributes to Rice stripe virus symptoms whereas osa-miR171b overexpression caused opposite effects, suggesting that expression of miR171-targeted mRNAs may facilitate viral infection [11]. In addition, miR171 has been suggested to be involved in the regulation of abiotic stresses based on its upregulation in Arabidopsis seedlings grown under high salinity, cold, and drought conditions [12].

Target mimic is a non-cleavable miRNA complementary sequence embedded within a longer endogenous or artificial RNA. In contrast to overexpression of a miRNA, which will silence its cognate mRNA targets and hence faithfully report on their functions, the target mimic acts as an “miRNA sponge” that titer out complementary miRNAs and hence is suitable for their functional characterization [13]. Furthermore, ectopic expression of miRNA or its cleavage-resistant mRNA target may lead to deceptive identification of miRNA function, due to incorrect spatio-temporal expression. Overexpression of miR171 resulted in various opposite phenotypes such as dwarfed barley with less tillers and taller rice with more tillers, and even silencing of miR171 can lead to different phenotypes between various species [14]. Ath-miR171a downregulation by single target mimic configurations resulted in a range of phenotypes consistent with ath-miR171a involvement in multiple developmental processes. Common phenotypes included closed buds and reduced pollination due to altered sepal development that bent the carpels, and pale green leaves due to reduced chlorophyll accumulation [15,16]. In addition, ath-miR171a-depeleted Arabidopsis had larger rosette leaves, a larger root system during the growth in soil and modified leaf angle under limited light conditions [16]. Nevertheless, a single target mimic sequence can only silence complementary miR171 members, but will not effectively bind miR171 members with sequence offset, thus limiting the efficacy of such configuration for functional analysis of miR171. Recently, expression of two target mimic sequences in a single transcript via short tandem target mimic (STTM) configuration was shown to efficiently induce complementary miRNAs degradation in Arabidopsis and tomato [13,17]. This approach, which may be applied to silence two different miRNAs in parallel, is thus highly suitable for the in planta functional characterization of miR171. Indeed, this approach has been successfully applied in rice. Rice STTM171 plants were semidwarf and had semienclosed panicles and drooping flag leaves. These unique phenotypes suggest divergent functions for miR171 in dicots and monocots [14,18].

Tomato is the number one non-starchy vegetable consumed worldwide and also serves as a primary model for fruit development and ripening. Nevertheless, at present, tomato miRNAs are poorly annotated and the functions of most remain elusive. To date, six miR171 members, sly-miR171a-f, were cloned from tomato (miRBase, release 22) [19]. Previously, we have demonstrated that sly-miR171a and sly-miR171b guide the cleavage of *SlHAM* and *SlHAM2* and sly-miR171b also guides the cleavage of the tomato *NSP2* homolog *SlNSP2L*. Sly-miR171a and sly-miR171b overexpression resulted in over-proliferation of meristematic cells in the periphery of meristems and in the organogenic compound leaf rachis, suggesting that sly-miR171-targeted *SlHAMs* function in meristem maintenance and compound leaf morphogenesis [6]. As part of our continuous effort to unravel the identity and roles of tomato miRNAs, in the current study, we re-annotated the tomato miR171 family and utilized the STTM approach to silence abundant members in the family and investigate their functions. Our results revealed the presence of a much more complex miR171 family than previously documented in tomato and its necessity for vegetative, anther, and pollen development.

## 2. Results and Discussion

### 2.1. The Tomato miR171 Family of miRNAs

**Figure 1:**
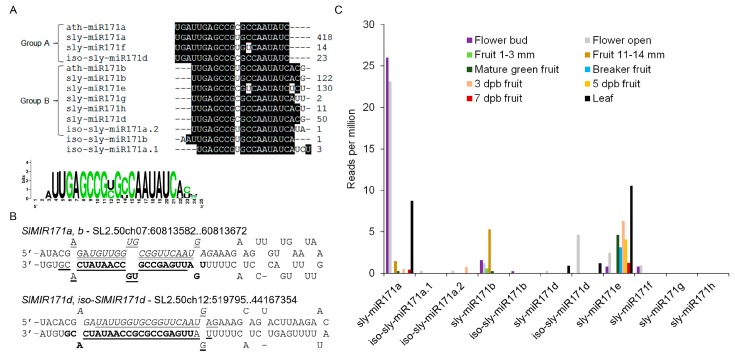
The tomato miR171 family. (**A**) Nucleotide sequence alignment of Arabidopsis (ath-miR171) and tomato (sly-miR171) miR171 members. Relative abundance in seedlings is indicated for each on the right. (**B**) Examples of sly-miR171 precursors that encode two sly-miR171 isoforms. The sequence of *SlMIR171a,b* (SL2.50ch07:60813582..60813672) and *SlMIR171d*, *iso-SlMIR171d* (SL2.50ch12:519795..519887) stem and loops. The sequences of sly-miR171a/d, sly-miR171a*/d*, sly-miR171b/ sly-miRiso-d and sly-miR171b*/iso-d* are bold-face, italicized, underlined and double-underlined, respectively. (**C**) Accumulation of sly-miR171 members in flower and fruit tissues of tomato cv. Microtome (Flower and fruit) and Heinz (leaf) based on small RNA-seq data deposited in the TFGD database. Dpb—days post breaker.

Six sly-miR171 members, sly-miR171a-f were previously cloned from tomato (miRBase, release 22). To identify additional sly-miR171 members, the small RNAs deposited in the Tomato Functional Genomics Database (TFGD; http://ted.bti.cornell.edu/cgi-bin/TFGD/sRNA/sRNA.cgi) and in-house tomato cv. M82 small RNA data [20] were queried with known miR171 sequences. This search detected eleven differentially abundant 21-nucleotide putative sly-miR171 sequences, including the previously cloned sly-miR171a, b, e, f and a 1-nucleotide longer version of sly-miR171d (Figure 1A). Sequence alignment revealed that identified sly-miR171 sequences can be divided into two groups, which are offset by three nucleotides relative to each other, similar to the Arabidopsis miR171 founder sequences ath-miR171a (group A) and ath-miR171c (group B). By mapping the putative sly-miR171 sequences to the tomato genome followed by alignment of the cloned small RNAs to their predicted pre-miRNAs sequences, we identified the miRNA stars (miRNA*) for all, indicating that they are authentic miRNAs (Table S1). In addition, this alignment revealed that one pre-miRNA (*SlMIR171a,b*) encodes for both sly-miR171a and sly-miR171b and their respective miRNA* strands (Figure 1B; Table S1). Moreover, four additional newly identified miR171 members (iso-sly-miR171a.1, iso-sly-miR171a.2, iso-sly-miR171b, iso-sly-miR171d) were found to be encoded by an identical precursor as that of other miR171 members and overlapped them in sequence (Figure 1B and Table S1), suggesting that they represent iso-miRNAs [21]. Querying the TFGD with these sequences confirmed their expression in tissues other than seedlings supporting their functionality as miRNAs (Figure 1C). It is noteworthy that iso-sly-miR171d miRNA* strand was previously annotated as sly-miR171c (miRBase, release 22). This analysis indicates that the tomato miR171 family is much more complex than previously thought, but it is of medium size compared to other miR171 families such as in *Glycine max* that contains up to 21 members (miRBase, release 22). Nevertheless, such complexity may hint on redundancy and specialization among different sly-miR171 members. Sly-miR171a and sly-miR171b, which represent group A and B sly-miR171 members, respectively, guide the cleavage of *SlHAM* and *SlHAM2*. Sly-miR171b, but not sly-miR171a, also guides the cleavage of the tomato *SlNSP2L* [6]. Prediction of mRNA targets and mining the published tomato degradome data [22] did not reveal strong evidence for additional sly-miR171-guided mRNA cleavage (Supplement 1).

To identify sly-miR171 sites of activity in tomato, analysis of public small RNAseq data at TFGD was performed. This analysis revealed that sly-miR171a, sly-miR171b, iso-sly-miR171d, and sly-miR171e are the most abundant members in the family, but the expression of each is prominent in a distinct tissue or developmental stage: sly-miR171a—leaves, floral buds and anthesis flowers, sly-miR171b—immature green fruit, iso-sly-miR171d—anthesis flowers and sly-miR171e—leaves, mature and ripening fruit (Figure 1C). Hence, the sly-miR171 family functions in vegetative as well as reproductive tissues with possible functional diversification between different members.

### 2.2. Knockdown of Sly-miR171 Activity Using the STTM Approach

**Figure 2:**
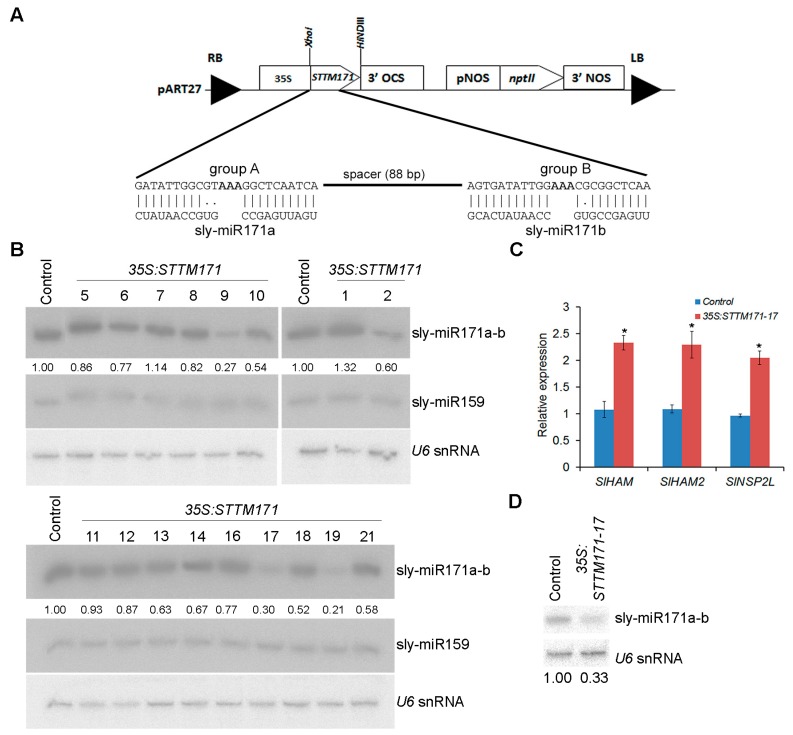
Generation of transgenic STTM171 tomato with reduced sly-miR171 levels. (**A**) A scheme of the Short Tandem Target Mimic construct used for tomato M82 transformation. The Watson–Crick pairings between group A and B target mimic sites and sly-miR171 representative members are shown in the expanded region. (**B**) RNA gel blot analysis of sly-miR171 levels in indicated transgenic T0 plants. Total RNA (5 µg) from leaves was probed by sly-miR171a (sly-miR171a-b), sly-miR159 and *U6* antisense probes. Sly-miR171 expression levels were determined after normalization to sly-miR159 and *U6* snRNA by geometric averaging and are indicated below. (**C**) RT-qPCR analysis of sly-miR171 target transcripts in RNA from young leaves of one-month old T2 35S:STTM171-17 plants. *TIP41* expression values were used for normalization. Error bars indicate ± SD of three biological replicates, each measured in triplicate. Asterisks indicate significant difference relative to *35:GFP* control plants (Tukey–Kramer multiple comparison test; *p* < 0.01). (**D**) RNA gel blot analysis of sly-miR171 in 5 µg total RNA from the samples analyzed in C. The blots were probed with sly-miR171a (sly-miR171a-b) antisense probe. Sly-miR171 expression levels were determined after normalization to *U6* snRNA and are indicated below.

Previously it was demonstrated that STTM configuration, which contains two target mimic sequences separated by a spacer, is very effective in counteracting the activity of several miRNAs in Arabidopsis and tomato [13,17]. Therefore, a similar STTM configuration was chosen to knockdown sly-miR171 family activity and uncover its importance for tomato development. Since group A and group B sly-miR171 members are offset by three nucleotides relative to each other, the STTM171 fragment was designed to comprise two different target mimic sequences, each of which have complementarity suitable to bind most of group A or group B sly-miR171 members, especially the most abundant sly-miR171 members (Figure 2A and Figure S1). The STTM171 fragment was cloned downstream of the CaMV *35S* promoter (*35S:STTM171*) and then transformed into tomato cv. M82. Seventeen independent transgenic *35S:STTM171* plants were regenerated and screened by northern blot for reduced sly-miR171a and sly-miR171b levels. This analysis identified three T0 primary transformants (9, 17, and 19) with significantly reduced sly-miR171 levels compared to control transgenic *35S:GFP* plants (Figure 2B). Plants *35S:STTM171-9* and *35S:STTM171-19*, which accumulated ~27% and ~21% of the total sly-miR171 levels, respectively, produced only few completely seedless fruits. Compared to the control plants, the *35S:STTM171-17* T0 plant, which accumulated ~30% of total sly-miR171 levels, produced smaller fruit in size and number, most of which were seedless and few contained a small number of seeds (Figure S2A,B). The smaller fruit size of *35S:STTM171-17* plants was probably a secondary effect which emerged due to the reduction in seed number [23]. Transformation efforts to produce additional independent fertile transgenic plants with significantly reduced sly-miR171 levels were not successful (data not shown). The sterility of *35S:STTM171-9* and *35S:STTM171-19* plants prevented further analysis of their progeny. Thus, further characterization of STTM171 plants was performed on *35S:STTM171-17* T2 and T3 progeny. Quantitation of sly-miR171-targeted *SlHAM*, *SlHAM2*, and *SlNSP2* transcripts in young leaves of the *35S:STTM171-17* T2 plants revealed significant ~2–2.5 fold upregulation in all (Figure 2C) indicating that both sly-miR171a and sly-miR171b activities were attenuated in these leaves by *35S:STTM171* expression. Indeed, quantitation of sly-miR171a-b in these leaves confirmed their reduced accumulation (Figure 2D).

### 2.3. Sly-miR171 Silencing Affected Compound Leaf Morphogenesis and Increased Branching

**Figure 3:**
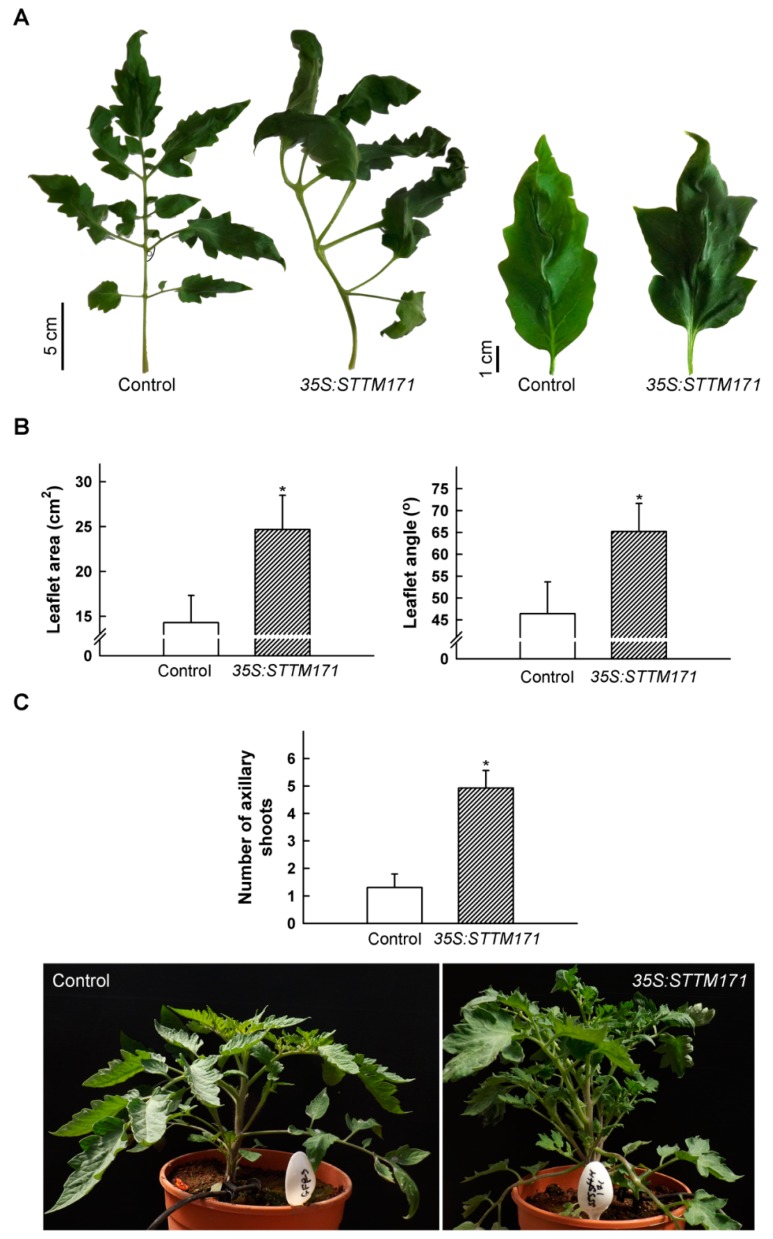
Vegetative phenotypes of *35S:STTM171* plants. (**A**) Photograph of representative fifth leaf and terminal leaflet from 45 DAG plants of indicated genotypes. (**B**) Quantitation of primary leaflet area and petiolule angle (indicated in (A)) in leaves (n = 26) similar to those shown in (A). (**C**) Quantitation of the number of axillary shoots on the main stem (≥0.5 cm) at eight leaf stage plants (n = 13). Error bars indicate ±SD. Asterisks indicate significant difference as determined by Student’s *t*-test (*p* ≤ 0.001). Representative plant of each genotype is shown below. Pot diameter = 18.8 cm.

During vegetative development the compound leaves of *35S:STTM171-17* plants developed primary leaflets that frequently had a distorted growth angle, were larger and their lobes were deeper than that of the control, implicating sly-miR171 in compound leaf morphogenesis (Figure 3A,B). In addition, compared to control plants, the number of axillary shoots was significantly higher in the *35S:STTM171* plants (Figure 3C). This phenotype is consistent with the increased lateral branch number observed in transgenic tomato plants that ectopically expressed *SlHAM2*/*SlGRAS24*, which was upregulated in *35S:STTM171* leaves (Figure 2C), and with transgenic Arabidopsis that ectopically expressed the ath-miR171c-ressitant versions of *SCL6-II*/*HAM1*, *SCL6-III*/*HAM2*, and *SCL6-IV*/*HAM4* [24,25]. Both *SlHAM* and *SlHAM2* are abundant in vegetative and reproductive meristems and function in their maintenance [6]. Thus, sly-miR171 may suppress lateral branching by the negative regulation of the expression of *SlHAM2* and apparently also *SlHAM* in axillary meristems.

### 2.4. Sly-miR171 Silencing Affected Pollen Morphology and Production

**Figure 4:**
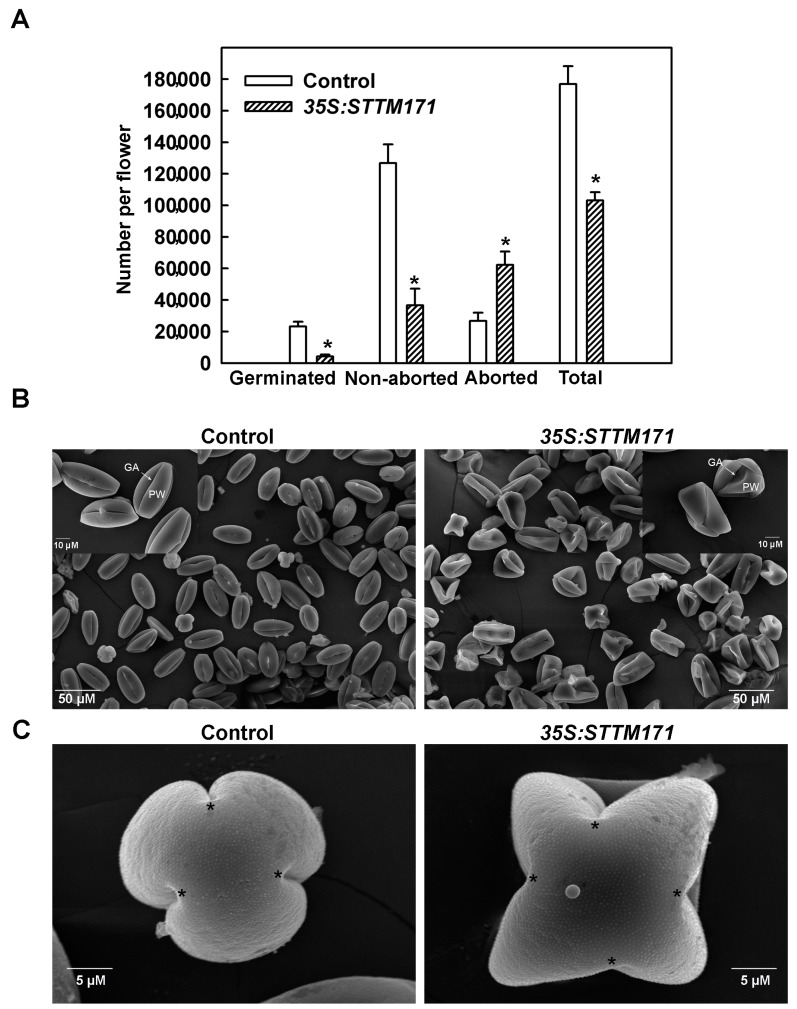
Effect of miR171 family downregulation on pollen quality and quantity. (**A**) Quantitation of total, aborted, non-aborted, and germinated mature pollen grains per anthesis flower of indicated genotype (n = 30). Asterisks indicate significant difference as determined by Student’s *t*-test (*p* ≤ 0.01). (**B**) Scanning electron micrographs of dehydrated pollen grains from indicated genotypes. Note the high number of collapsed pollen grains in the *35S:STTM171* sample. Inset shows magnified views of few representative pollen grains from each genotype. PW: pollen wall; GA: germinal aperture. (**C**) Scanning electron micrographs of polar view of representative mature pollen grains from indicated genotypes. The locations of the germinal aperture are indicated by asterisks.

Despite the normal number and morphology of their flowers, *35S:STTM171* plants set only few fruits that were mostly seedless. Whereas manual pollination of *35S:STTM171* flowers with wild-type pollen rarely succeeded, the reciprocal pollination completely failed to induce fruit set, indicating reduced male fertility of the *35S:STTM171* flowers. A similar male sterile phenotype was observed in the transgenic F1 progeny from the cross between wild type and *35S:STTM171* T2 plants suggesting that the *35:STTM171* transgene caused the phenotype. This is also supported by the finding that overexpression of the sly-miR171 target *SlHAM2*/*SlGRAS24* reduced seed number due to male sterility [25]. To further understand the basis of the male sterility phenotype, we assessed the productivity and quality of pollen grains in anthesis flowers of *35S:STTM171* by differential Alexander staining [26], which distinguishes between aborted and non-aborted pollen, and by testing pollen germination. This analysis indicated that the average total number of *35S:STTM171* pollen grains was reduced by 42% compared to control flowers. Moreover, the majority of the *35S:STTM171* pollen grains were aborted (60.4%), a fraction that is 4-fold higher than that found in control pollen grains. Consistent with that, the number of germinated pollen grains fell by 5.6-fold (Figure 4A). These data suggest that the *35S:STTM171* plants produce relatively small numbers of poor-quality pollen grains compared to control. This is consistent with the apparent sterility of *35S:STTM171* plants and explains why manual fertilization of wild-type tomato flowers with *35S:STTM171* pollen grains was unsuccessful. The observed almost seedless fruit and reduced pollen viability phenotypes in *35S:STTM171* plants are reminiscent to those described in *SlGRAS24*/*SlHAM2*-overexpressing plants [25]. This similarity suggested that the negative regulation of *SlHAM2* levels by sly-miR171 may be critical for pollen development.

**Figure 5:**
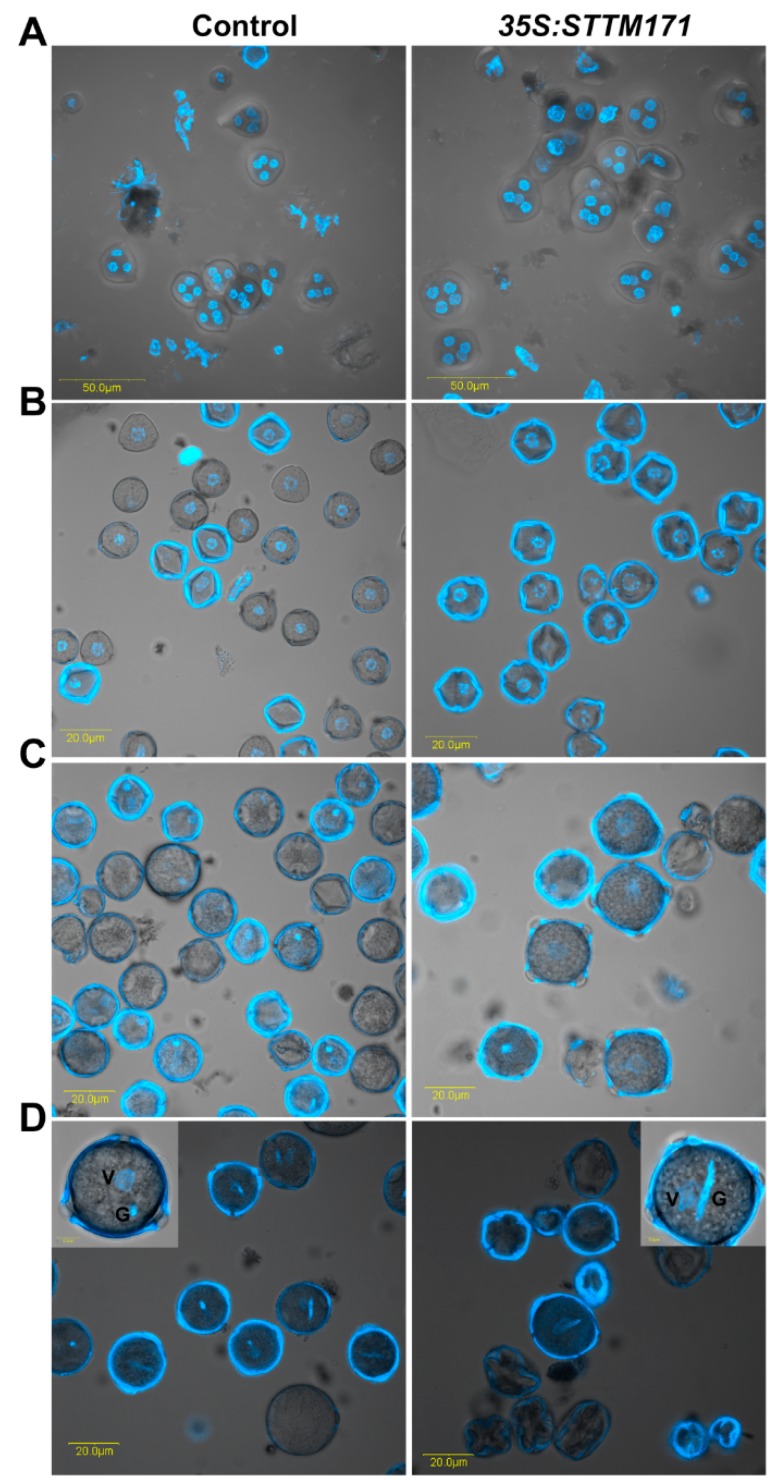
The *35:STTM171* pollen grains contain normal germ unit. DAPI fluorescence micrographs of control and *35:STTM171* tetrads (**A**), microspores (**B**), binucleate microspore (**C**), mature pollen grains (**D**). Inset in (D) shows magnified view of a representative pollen grain. The vegetative (V) and generative (G) cells are indicated. Note that the pollen surface fluorescence is due to auto-fluorescence at the same wavelength used for DAPI detection.

To determine the cause of pollen abortion in *STTM171* expressing plants we initially analyzed pollen morphology by scanning electron microscope (SEM). Wild-type tomato cv. M82 mature pollen grains are psilate and tricolporate [27]. SEM analysis of *35S:STTM171* mature pollen grains revealed that although they remained psilate, they had deformed shapes. These included a collapsed wall, disordered germinal apertures and instead of being tricolporate many were tetracolpate (Figure 4B,C). In contrast to their morphological abnormalities, DAPI staining of *35S:STTM171* developing pollen nuclei did not reveal any nuclear aberrations during the formation of tetrads (Figure 5A), microspores (Figure 5B,C), and bicellular pollen (Figure 5D), suggesting that morphological and not nuclear aberrations underlie the poor quality of the *35S:STTM171* pollen.

### 2.5. The 35S:STTM171 Anthers Accumulated Reduced Sly-miR171 Levels Associated with Delayed Tapetum Degeneration and Reduced Callose Deposition

Male sterility is frequently associated with deviations in the development of the anthers that contain the sporogenous tissue and its circumjacent tissues, the tapetum and middle layer, which ultimately gives rise to the pollen grains and support pollen development correspondingly [28]. In situ of miR171 in *N. benthamiana* developing flowers has detected high uniform expression in the pollen sacs and surrounding tissues of young anthers [9], suggesting miR171 involvement in pollen development. Northern analysis with sly-miR171a/b/e validated probes (Supplement 2; Figure S3) showed that sly-miR171a/b and sly-miR171e are abundant in anthers throughout their development until maturity (anthesis flower) (Figure S4A). Anther developmental stages were defined according to the study of flower development of tomato by Brukhin et al. [29] and verified by DAPI staining from the tetrad stage (Figure 5). In agreement with the northern analysis, deep sequencing of small RNAs from developing tomato anthers identified sly-miR171a, its group member iso-sly-miR171d and sly-miR171d, a group member of sly-miR171e [30]. Compared to control anthers, the anthers of *35S:STTM171* accumulated significantly reduced levels of sly-miR171a/b in the meiosis (4 mm, stage 9), tetrad stage (5 mm, stages 10–11), free microspores stage (6 mm, stage 12) and mature pollen stage (12 mm, stages 18–19). Moreover, reduced levels were observed for sly-miR171e for which silencing was even more pronounced than for sly-miR171a/b. Depending on the developmental stage, the *35S:STTM171* anthers accumulated only around 5–20% of the control levels of sly-miR171e (Figure S4B).

**Figure 6:**
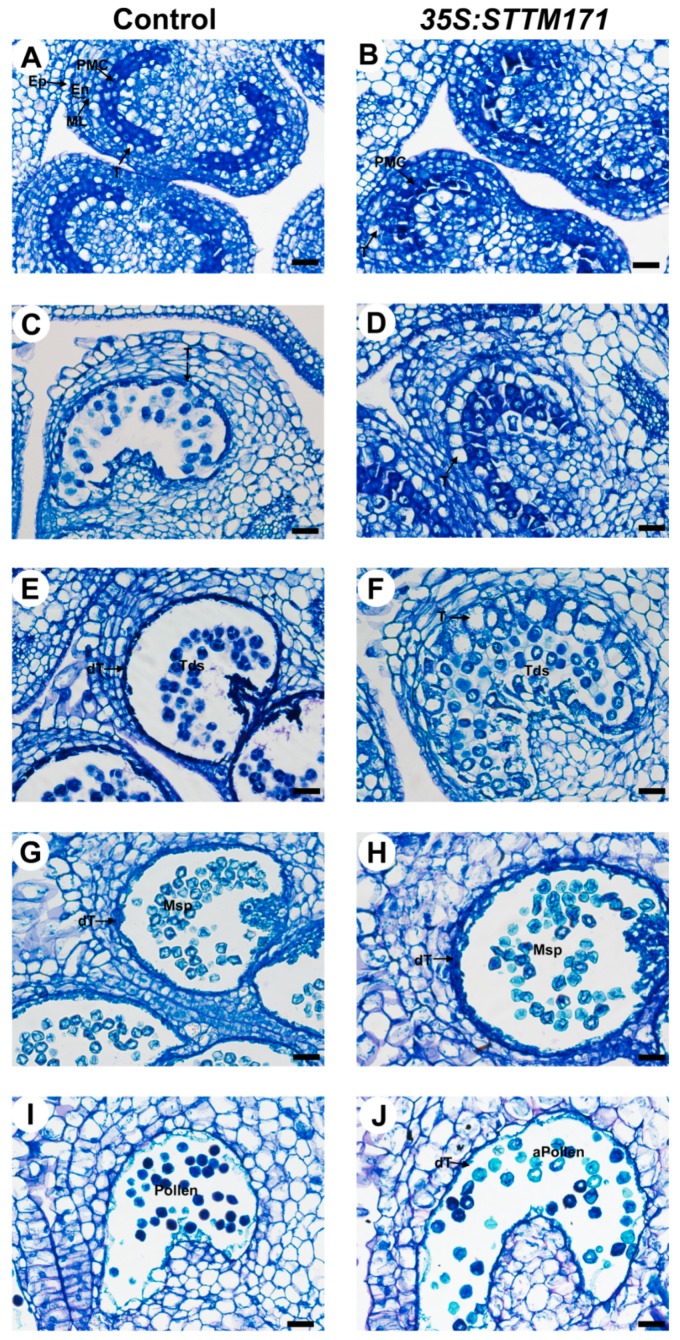
Histological analysis of control and *35S:STTM171* anthers. Pictures of Toluidine-blue stained cross sections of control and *35:STTM171* anthers at subsequent stages of microspore development as follows: (**A**,**B**) microsporocyte stage, (**C**,**D**) meiosis stage, (**E**,**F**) tetrad stage, (**G**,**H**) microspore stage, (**I**,**J**) bicellular pollen stage. dT-degenerated tapetum; En-endothecium; Ep-epidermis; ML-middle cell layer; Msp-microspore; MMC-microspore mother cell; T-tapetum; Tds-tetrads; aPollen-aborted pollen. Scale bars = 20 μm.

Next, we asked whether the reduced accumulation of sly-miR171 in *35S:STTM171* anthers is associated with abnormal development of anther tissues. To answer that, control and *35S:STTM171* anthers at major developmental stages were comparatively examined using transverse section light microscopy. Following examination of anthers under light microscopy showed that at the microsporocyte stage (3 mm buds, stage 8) the control pollen mother cells (PMC) are enclosed by a single layered tapetum (Figure 6A). At this stage, no distinct differences between control and the *35S:STTM171* transgenic anthers were observed (Figure 6B). Morphological differences were initially observed at the meiosis stage (4 mm bud, stage 9, Figure 6C). At that stage the control tapetal layer, which is composed from condensed tapetum cells, as indicated by their shrinkage and deep staining, encloses dividing microsporocytes, whereas in *35S:STTM171* anthers, tapetal cells remain expanded and vacuolated and dividing microsporocytes were not observed (Figure 6D). At the tetrad stage control tapetal cells were completely shrunk (5 mm bud, stage 11, Figure 6E), likely due to the initiation of programmed cell death (PCD) [31], and enclosed separated tetrads. In contrast, in *35S:STTM171* anthers, tapetal cells were not shrunken and instead were enlarged while most tetrads were not separated (Figure 6F). At the microspore stage, degenerated tapetal layer enclosing free microspores was observed in both control (8 mm bud, stages 12–13, Figure 6G) and *35S:STTM171* anthers (Figure 6H), except that in the latter the tapetum was less degenerated. At the bicellular pollen stage the control tapetum was completely degenerated and pollens were mature with characteristic densely stained cytoplasm (10 mm bud, stage 17–18, Figure 6I), whereas in *35S:STTM171* anthers, remnants of the degenerated tapetum were still visible and enclosed aborted pollen (Figure 6J). These observations indicated that the degeneration of the tapetum in the anthers silenced for sly-miR171 was delayed and initiated only after the tetrad stage in comparison to control where tapetum degradation occurred significantly earlier and on time.

Whereas tapetum is still intact at the tetrad stage, its cells export sporopollenin on microspore primexine surface that will provide a basis for future assembling of lipid materials into the pollen coat exine. During this process and earlier meiosis, the PMC and tetrads are surrounded by callose layer that provides protection for developing microspores. Later, the callose layer goes through degradation by tapetum supplied callase and the tapetum goes through PCD aiming to supply additional materials to the microspores [32,33,34]. The degradation of callose allows microspore release from tetrads and degradation of the tapetum supplies lipidic tapetum-derived materials for pollen exine and nutrients for pollen maturation [33,34]. The timing of tapetal cell death and consistency of the above-described events are critical for pollen development and interference in this process usually results in male sterility [35]. In several studies, delaying tapetum degeneration was shown to affect pollen morphology and results in pollen abortion. The rice mutant *tapetal degeneration retardation* (*Ostdr*) shows delayed tapetal breakdown resulting in a failure of pollen wall deposition and subsequent microspore degeneration [36]. Mutation in rice *OsACOS12* delays PCD-induced tapetum degradation leading to collapsed aborted pollen [37]. Thus, a likely possibility is that the delayed degeneration of *STTM171* expressing tapetum may be responsible at least in part for the deformed morphology of respective pollen grains.

**Figure 7:**
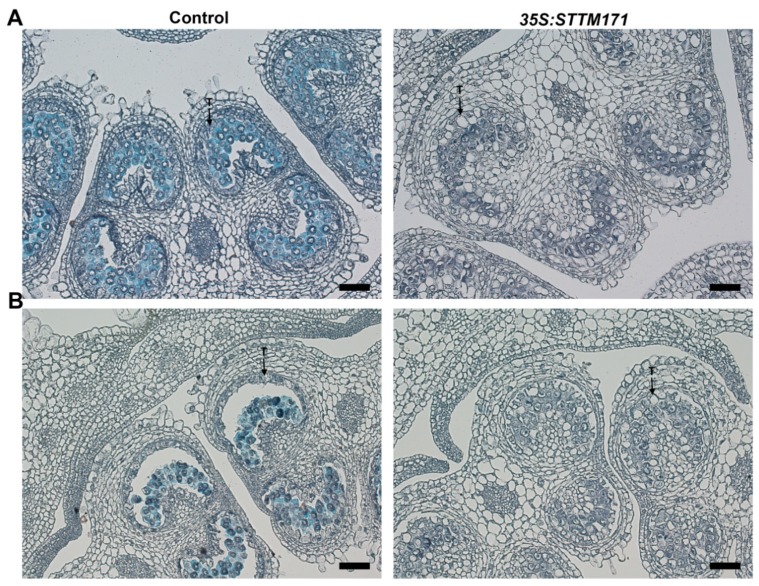
Callose detection in control and *35S:STTM171* anthers. Pictures of Lacmoid stained cross sections of anthers at the meiosis (**A**, 4 mm bud) and tetrad (**B**, 5 mm bud) stages are shown. T-tapetum. Scale bars = 20 μm.

Callose (β-1,3 glucan) protects PMC and later developing microspores from swelling, rupture, impact of diploid tissues and serves as a mold for future exine layer [38,39]. Often, defective tapetum development plan is accompanied with depletion of callose. To test if the delayed degeneration of *35S:STTM171* tapetum cells affected callose dynamics we performed a lacmoid stain of control and of *35S:STTM171* anthers at the meiosis and tetrad stages. We observed strong staining in control anthers at the meiosis stage (Figure 7A) and much weaker staining at the tetrad stage (Figure 7B) probably as a result of initiated callose degradation by tapetum supplied callase. In contrast, significantly weaker staining was observed in the *35S:STTM171* anthers (Figure 7A,B). Moreover, the weak callose staining was associated with enlarged tapetum cells characteristic of those that have not initiated PCD, rather than condensed cells undergoing PCD, as in the wild-type (Figure 6D,F and Figure 7). In transgenic tobacco plants with delayed tapetum development, male sterility was caused because of premature degradation of callose [40]. In *DISFUNCTIONAL TAPETUM1* mutant enlarged tapetum cells and thin callose layer caused pollen collapse which resulted in male sterility [41]. This suggests that the delayed tapetum development in *35S:STTM171* anthers also delayed the callose deposition. Alternatively, callose depletion might be caused independently of tapetum development as in the *CALLOSE SYNTHASE5* mutants, where failure to produce callose resulted in collapsed pollen [33,42] reminiscent of the *35S:STTM171* pollen phenotype. Taken together these results suggest that the silencing of sly-miR171 in the *35S:STTM171* anthers perturbed tapetum development, callose dynamics and as a result pollen ontogenesis. However, additional studies are required to support this suggestion and determine the mechanism by which these miR171 members regulate anther development. 

## 3. Materials and Methods

### 3.1. Plant Material and Growth Conditions

Tomato cv. M82 plants and seedlings were grown under greenhouse and growth chamber conditions, respectively, as previously described [20].

### 3.2. Plasmid Construction

The pART27-OP:SlMIR171a and pART27-OP:SlMIR171b responder plasmids were described elsewhere [6]. For the pART27-OP:SlMIR171e responder construct, a 239 bp fragment from *SlMIR171e* including the *pre-miR171e* was amplified with Xho_MIR171e_F and Hind_MIR171e_R which contained *Xho*I and *Hind*III sites at their 5’ ends (for primer sequences, see Supplementary Table S3 online). The amplified fragment was restricted with *Xho*I and *Hind*III and ligated into pART27 binary vector containing the OP array to obtain pART27-OP:SlMIR171e [6]. For the *35S:STTM171* construct, a 136 bp STTM fragment was synthetically synthesized and then PCR amplified with Xho_STTM_171_F and Hind_STTM_171_R primers that contained *Xho*I and *Hind*III sites at their 5′ ends. The amplified fragment was restricted with *Xho*I and *Hind*III and cloned into the appropriate sites of the pART27 binary vector containing the CaMV 35S promoter and *Agrobacterium tumefaciens* octopine synthase terminator (OCS) [17] to obtain pART27-35S:STTM171.

### 3.3. Transformation of Tomato Plants

The binary vector pART27-35S:STTM171 was transformed into tomato cv. M82 and transgenic plants were selected as described previously [20]. Each kanamycin resistant plant was also subjected to genomic DNA PCR with the primer pair Xho_STTM_171_F and OCS_rev to detect the 35S:STTM171 transgene.

### 3.4. Total RNA Extraction and RNA Gel-Blot Analysis

Total RNA was extracted from different tomato tissues with Bio-Tri RNA reagent (Bio-Lab, Jerusalem, Israel) according to the manufacturer’s protocol. A small-RNA gel-blot analysis of the total RNA was performed as described previously [43] using a complementary radiolabeled oligos as probes (probe sequences are listed in Supplementary Table S3 online).

### 3.5. cDNA Synthesis and Quantitative RT-PCR Assay

First-strand cDNA was synthesized from 2 µg of total RNA with Maxima first strand cDNA synthesis kit (Thermo Scientific, Waltham, MA, USA) following the manufacturer’s instructions. A negative control (-RT) was used to ensure the absence of genomic DNA template in the samples. Three independent biological replicates were used for each sample, and quantification was performed in triplicate. PCR was performed in StepOnePlus Real-Time PCR System (Thermo Fisher Scientific) following the manufacturer’s instructions. Primer sequences are listed in Supplementary Table S3 online. Relative expression levels were normalized to *SlTIP41* as a reference gene, and calculated by the standard curve method.

### 3.6. Analysis of Leaf Morphology and Axillary Shoot Number

Primary leaflet area was calculated by Tomato Analyzer 3.0 software [44]. Leaflet angle, namely the angle between the petiolule and the rachis was measured manually by a protractor. The number of axillary shoots (≥5 mm long) was counted at eight-leaf stage tomato plants.

### 3.7. Determination of Pollen Quality and Quantity

To determine the pollen quantity and quality, mature pollen was extracted, stained, and counted according to Firon et al. [45]. Briefly, two flowers at the day of anthesis were sampled from control and transgenic plants and three anthers were removed from each flower, sliced in the middle, and immediately placed in a microcentrifuge tube containing germination solution (0.5 mL, 10% sucrose, 2 mM boric acid, 2 mM calcium nitrate, 2 mM magnesium sulfate, and 1 mM potassium nitrate). Then the pollen grains were released by vortex, incubated for 4 h at 25 °C, and stained with Alexander dye, that colors aborted pollen grains in blue-green, and non-aborted pollen grains in magenta-red [26]. The pollen grains were counted under a light microscope in a haemocytometer, eight fields for each sample.

### 3.8. Scanning Electron Microscopy (SEM) and 4′,6-Diamidino-2-Phenylindole (DAPI) Staining of Pollen Grains

For SEM analysis, pollen grains were collected and placed in FAA (3.7% formaldehyde, 5% acetic acid, 50% EtOH) solution until use. Then the FAA solution was removed and pollen grains were dehydrated in an increasing gradient of ethanol (up to 100%), critical-point-dried, mounted on a copper plate and gold-coated. Samples were viewed in a Jeol 5410 LV microscope (Tokyo, Japan). To stain pollen grains with DAPI, grains at different developmental stages were released by vortex into a DAPI solution (0.1 M sodium phosphate buffer (pH 7), 1 mM EDTA, 0.1% Triton X-100, 0.4 µg/mL DAPI), incubated for 10 min at room temperature and then viewed by Olympus IX81/FV500 laser-scanning confocal microscope (Olympus) at 361 nm maximum absorption, 461 nm maximum emission.

### 3.9. Histology and Callose Staining

For histological analyses, stamens at different developmental stages were taken and fixed in FAA solution until use, then dehydrated in increasing concentrations of ethanol (70%, 80%, 90%, 95%, and 100%), cleared with histoclear, and embedded in paraffin. Microtome-cut sections (6-µm thick) were spread on microscope slides, and stained with 0.03% Toluidine blue O. For callose staining microtome-cut sections of stamens were stained with 0.2% Lacmoid in 50% ethanol for 48h, then placed in 1% sodium bicarbonate in 50% ethanol for 10 min [46]. Stained slides were examined under bright-field using an Olympus (Olympus, www.olympus-lifescience.com) light microscope equipped with a digital camera.

## Supplementary Materials

The following are available online at http://www.mdpi.com/2223-7747/8/1/10/s1, Supplement 1. Prediction of target mRNAs for sly-miR171 and corresponding sly-mi171* strands. Supplement 2. Validation of probe specificity of sly-miR171. Figure S1. Alignment between STTM171 and sly-miR171 sequences. Figure S2. Fruit of *35:STTM171* T2 plants. Figure S3. Determination of the specificity of sly-miR171a, b, e RNA gel blot probes. Figure S4. Developing *35:STTM171* anthers accumulate reduced levels of sly-miR171. Table S1: A list of sly-miR171 precursors and corresponding miRNA/miRNA* pairs. Table S2: psRNATarget analysisa of sly-miR171 members and their star strands. Table S3: Primers and probes used in this study.
