# Supplementary material for: Functional Characterization of microRNA171 Family in Tomato
Source: Plants (Basel). 2019 Jan 4;8(1):10. doi: 10.3390/plants8010010 (PMC6358981; doi:10.3390/plants8010010)
Supplement: Supplementary file 1 [file plants-08-00010-s001.zip › plants-414116-supplementary-final/plants-414116-suppl-proofed.docx]

**Supplementary information for**

Functional characterization of microRNA171 family in tomato

**Michael Kravchik^1^, Ran Stav^1^, Eduard Belausov^1^ and Tzahi Arazi^1,^***

^1^ Institute of Plant Sciences, Agricultural Research Organization, Volcani Center, P.O. Box 6, Bet Dagan 50250, Israel; [michael.kravchik@mail.huji.ac.il](mailto:michael.kravchik@mail.huji.ac.il) (KM); [ranstav@volcani.agri.gov.il](mailto:ranstav@volcani.agri.gov.il) (SR) [eddy@volcani.agri.gov.il](mailto:eddy@volcani.agri.gov.il) (BE)

* Correspondence: [tarazi@agri.gov.il](mailto:tarazi@agri.gov.il); Tel.: +972-3-968-34-98

Supplement 1

*Prediction of target mRNAs for sly-miR171 and corresponding sly-mi171* strands*

It was suggested that ath-miR171a* acts as functional miRNA and guides the cleavage of *SU(VAR)3-9 HOMOLOG8* in *A. thaliana* [1]. To check whether the newly identified sly-miR171 members may target additional mRNAs and the possibility that certain sly-miR171* may act as a miRNA, we used the psRNATarget web tool (schema V2, 2017 release) [2] to predict their putative target mRNAs from the tomato genome cDNA database (ITAG release 3.1). This analysis predicted that *Solyc02g086540*, which encode a putative acetyl glucose aminyl transferase, may serve as potential target for iso-sly-miR171b and *Solyc06g076250*, *Solyc02g089263* and *Solyc01g110220* may serve as potential targets for iso-sly-miR171a.1*, iso-sly-miR171a.1* and sly-miR171e*, respectively, (Table S2). However, *Solyc06g076250* and *Solyc02g089263* are predicted to be inhibited by translational inhibition rather than cleavage, which is consistent with the lack of evidence for their miRNA-guided cleavage in the published tomato degradome data [3]. Moreover, the tomato degradome data did not contain evidence for the miRNA-guided cleavage of the rest of the predicted targets thus weakening the authenticity of these targets.


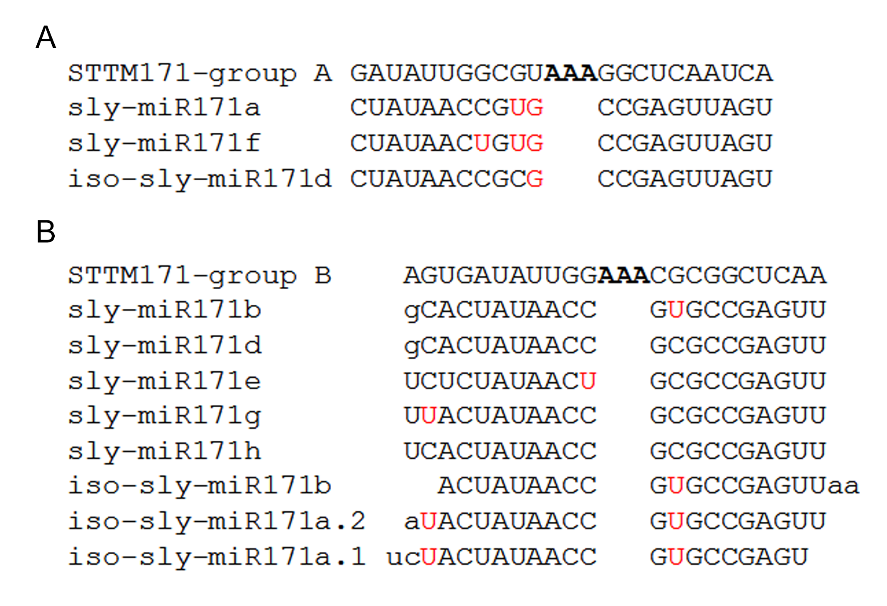


**Fig. S1.** Alignment between STTM171 and sly-miR171 sequences. Manual sequence alignment between STTM171 target mimic sequences and the respective group A (A) and group B (B) sly-miR171. Partial and no complementarity between the sly-miR171 and the target mimic sequence are indicated by red and lower case nucleotides, respectively. For convenience the sly-miR171 sequences are reversed (3'->5').


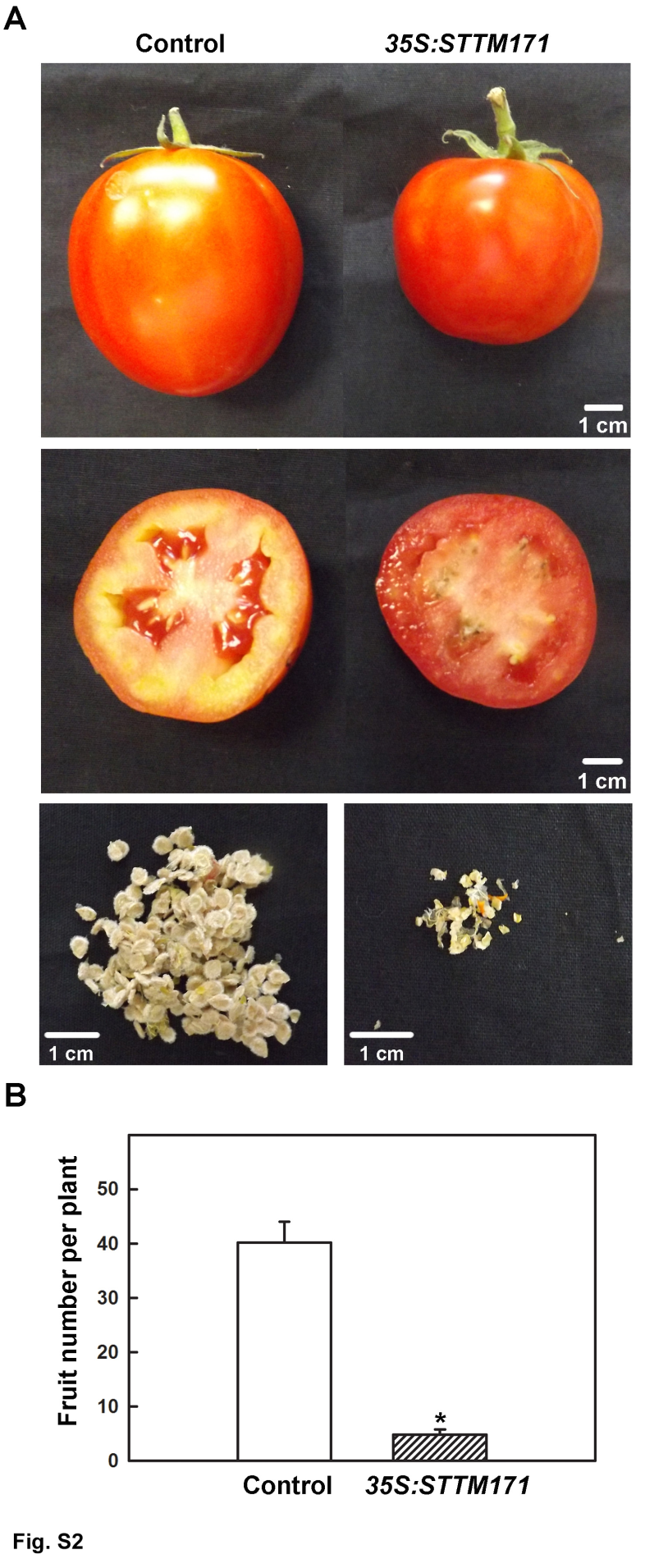


**Figure S2.** Fruit of *35:STTM171* T2 plants. (A) Whole (upper panel) and transverse (lower panel) section through control and *35:STTM171* fruit. Seeds extracted from identical fruit are shown below. (B) Quantitation of fruit number per plant. Error bars indicate ±SD (n = 13). Asterisks indicate significant difference as determined by Student’s t-test (P ≤ 0.01).

**Supplement 2**

*Validation of probe specificity of sly-miR171*

Sly-miR171a and sly-miR171b have a stretch of 18 identical nucleotides between them while sly-miR171e has two mismatches in the middle of that stretch (Fig. S1). Such high similarity called for validation of probe specificity which was conducted by RNA gel blots of transiently expressing corresponding precursors in *Nicotiana* *benthamiana* leaves with different probes. *A.* *tumefaciens* strain GV3101 cultures harboring the binary plasmids pART27-OP:SlMIR171a, pART27-OP:SlMIR171b, pART27-OP:SlMIR171e, were mixed with GV3101 culture harboring the binary plasmid pART27-35S:LhG4 to a final A_600_ of 0.5 and infiltrated into young leaves of 3-week-old greenhouse-grown *N. benthamiana* plants. For small RNA blot analysis, leaf patches were collected 2-3 days post-infiltration, total RNA was extracted and subjected to RNA gel blot with oligo probes corresponding to sly-miR171a, sly-miR171b and sly-miR171e. As shown in Fig. S3, the sly-miR171a probe could detect sly-miR171b, although to a weak extent, and the sly-miR171b probe cross-hybridized with sly-miR171a completely, whereas the sly-miR171e probe did not hybridize with sly-miR171a or sly-miR171b. Therefore, the sly-miR171a probe was used to detect A and B clade members, and the sly-miR171e probe was used to probe sly-miR171e expression in further experiments. Ultimately, they were able to recognize most of the members of sly-miR171 family, because of high similarity within the miR171 family.


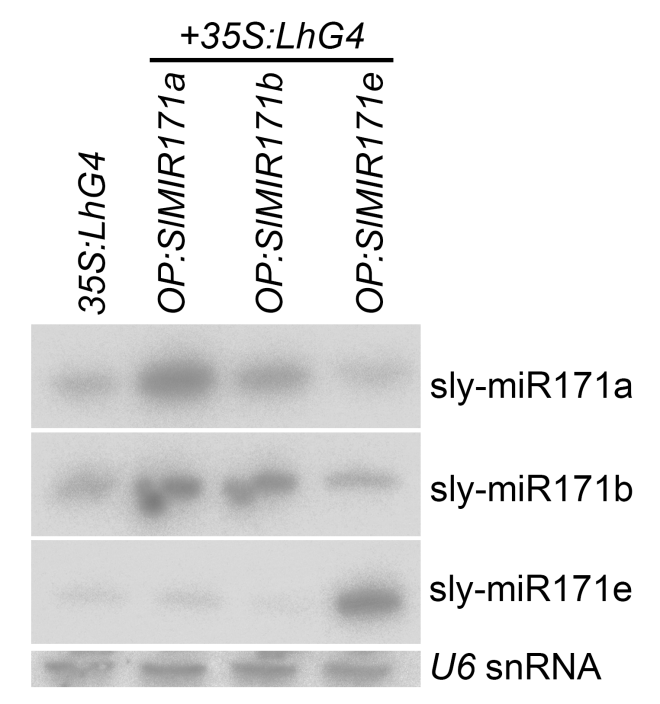


**Fig. S3.** Determination of the specificity of sly-miR171a, b, e RNA gel blot probes. *N. benthamiana* leaves were infiltrated with *A. tumefaciens* mixtures harboring the driver plasmid pART27-35S:LhG4 alone or with the indicated sly-miR171 responder plasmid (pART27-OP:sly-miR171) and total RNA (5 µg) from 2 days post infiltration leaves was subjected to RNA gel blot analysis with the indicated sly-miR171 antisense probe. The *U6* snRNA served as RNA loading control.


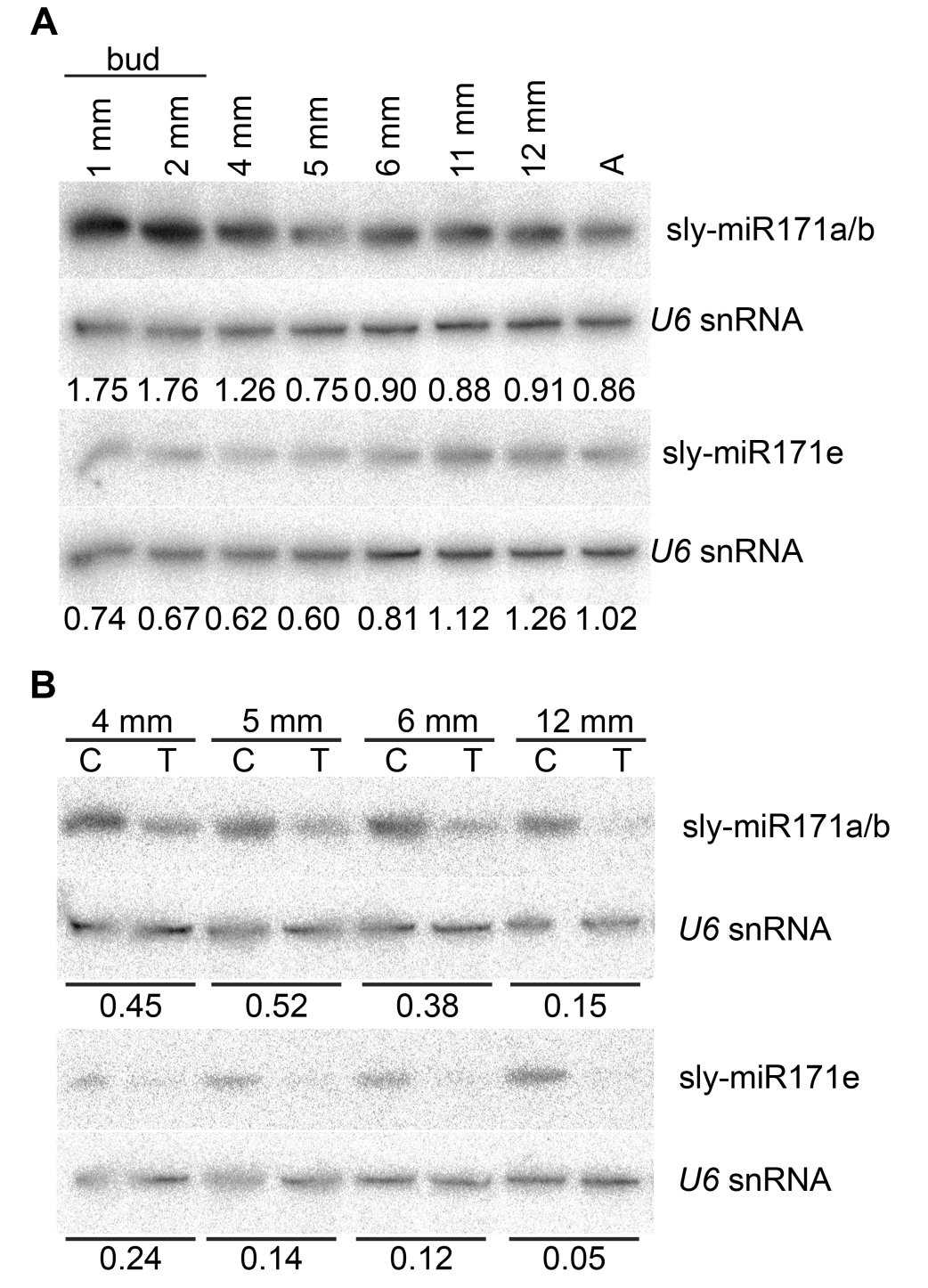


**Fig. S4.** Developing *35:STTM171* anthers accumulate reduced levels of sly-miR171. RNA gel blot analysis of sly-miR171 levels in (A) control and (B) *35:STTM171* anthers. Total RNA (5 µg) from isolated floral organs and anthers (anther developmental stage was defined by floral bud length) was probed by sly-miR171a (sly-miR171a-b) or sly-miR171e antisense probe. Sly-miR171 expression levels were determined after normalization to *U6* snRNA and in (B) also relative to control and are indicated below each panel. A – anthesis flowers, C – control, T – *35:STTM171*.

References

1. Manavella, P.; Koenig, D.; Rubio-Somoza, I.; Burbano, H.A.; Becker, C.; Weigel, D. Tissue-specific silencing of Arabidopsis thaliana SUVH8 by miR171a star. *Plant Physiol.* **2012**, 161, 805-812.

2. Dai, X.; Zhao, P.X. psRNATarget: a plant small RNA target analysis server. *Nucleic Acids Res.* **2011**, *39*, W155–W159.

3. Karlova, R.; van Haarst, J.C.; Maliepaard, C.; van de Geest, H.; Bovy, A.G.; Lammers, M.; Angenent, G.C.; de Maagd, R.A. Identification of microRNA targets in tomato fruit development using high-throughput sequencing and degradome analysis. *J. Exp. Bot.* **2013**, *64*, 1863–1878.
