# Supplementary material for: Functional Characterization of microRNA171 Family in Tomato
Source: Plants (Basel). 2019 Jan 4;8(1):10. doi: 10.3390/plants8010010 (PMC6358981; doi:10.3390/plants8010010)
Supplement: Supplementary file 1 [file plants-08-00010-s001.zip › plants-414116-supplementary-final/Table S1.docx]

| **Table S1.** A list of sly-miR171 precursors and corresponding miRNA/miRNA* pairs | | | | | | | |  |  |
| --- | --- | --- | --- | --- | --- | --- | --- | --- | --- |
| ^a^ small RNA numbers were determined by deep sequencing of 3-weeks old M82 tomato seedlings | | | | | | | |  |  |
| ^b^ Pre-miR171 secondary structures were predicted by RNAfold (M. Zuker, P. Stiegler (1981) Optimal computer folding of large RNA sequences using thermodynamic and auxiliary information, Nucl Acid Res 9: 133-148) | | | | | | | | | |
| ^c^Sum of all pre-miR171a numbers | | | | |  |  |  | | |
| ^d^Sum of pre-miR171a (SL2.50ch02:46752782..46752903) and pre-miR171f numbers | | |  |  | | | | | |
| ^e^Sum of pre-miR171b and pre-miR171a, b numbers | |  |  |  | | | | | |
| ^f^Sum of pre-miR171g and pre-miR171h numbers | |  |  |  | | | | | |
|  |  |  |  |  | | | | | |
| **Precursor/miRNA name** | **Precursor position** | **Strand** | **Clone numbers** | **Precursor sequence, secondary structure and associated miRNA/miRNA*** | | | | | |
| pre-miR171a | SL2.50ch02:46752782..46752903 | minus | - | UGAAACAGUAACUUUGAUAUUGGCCUGGUUCACUCAGACAACAAAAUGUAAACUAUAUUUGAAUGGUUGAGUUUUCGUUUUUCGUUUGAUUGAGCCGUGCCAAUAUCUCAGUUUCUCUUUCA | | | | | |
|  |  |  |  | .((((.((.((((..(((((((((.(((((((.((((((((((((((...((((((......))))))..))))).)))....)))))).))))))).)))))))))..)))).)).)))). | | | | | |
| sly-miR171a |  |  | 418^c^ | ......................................................................................UGAUUGAGCCGUGCCAAUAUC............... | | | | | |
| sly-miR171a* |  |  | 287^d^ | .................UAUUGGCCUGGUUCACUCAGA.................................................................................... | | | | | |
|  |  |  |  |  | | | | | |
| pre-miR171a | SL2.50ch03:61425171..61425271 | minus | - | GUAUAGUAAAAUGAUGUUGGAAUGGCUCAAUCAAAUCAAAUUUCUCAAAGUAUUGGGUCAUUUCAUUUGAUUGAGCCGUGCCAAUAUCAUCUUACUUAUAU | | | | | |
|  |  |  |  | (((((((((.((((((((((.(((((((((((((((.((((..(((((....)))))..)))).))))))))))))))).)))))))))).)))).))))) | | | | | |
| sly-miR171a |  |  | 418^c^ | ...................................................................UGAUUGAGCCGUGCCAAUAUC............. | | | | | |
| sly-miR171a* |  |  | 1 | ..............UGUUGGAAUGGCUCAAUCAAA.................................................................. | | | | | |
| iso-sly-miR171a.1 |  |  | 3 | .......................................................................UGAGCCGUGCCAAUAUCAUCU......... | | | | | |
| iso-sly-miR171a.1* |  |  | 32 | ..........AUGAUGUUGGAAUGGCUCAAU...................................................................... | | | | | |
|  |  |  |  |  | | | | | |
| pre-miR171a | SL2.50ch06:42762054..42762133 | plus | - | GAUGUUGGUGUGGUUCAAUUAGAUAACAAUCUCCACGUAAAAUUAUAGAGUUUGUUAUUUGAUUGAGCCGUGCCAAUAUC | | | | | |
|  |  |  |  | ((((((((..(((((((((((((((((((.(((..............))).)))))))))))))))))))..)))))))) | | | | | |
| sly-miR171a |  |  | 418^c^ | ...........................................................UGAUUGAGCCGUGCCAAUAUC | | | | | |
|  |  |  |  |  | | | | | |
| pre-miR171a | SL2.50ch06:44420862..44420963 | plus | - | AGGAGUUGGUGUGAUGUUGGAAUGGCUCAAUCUAAUGAAAGUUUCCAAACAUUUUUGGUCAUUUUAAUUUGAUUGAGCCGUGCCAAUAUCAUAUCGUCUUUU | | | | | |
|  |  |  |  | (((((.((((((((((((((.(((((((((((.(((.(((((..(((((....)))))..))))).))).))))))))))).)))))))))))))).))))) | | | | | |
| sly-miR171a |  |  | 418^c^ | .....................................................................UGAUUGAGCCGUGCCAAUAUC............ | | | | | |
| iso-sly-miR171a.2 |  |  | 1 | ........................................................................UUGAGCCGUGCCAAUAUCAUA......... | | | | | |
| iso-sly-miR171a.2* |  |  | 6 | ...........UGAUGUUGGAAUGGCUCAAUC...................................................................... | | | | | |
|  |  |  |  |  | | | | | |
| pre-miR171a, b | SL2.50ch07:60813582..60813672 | plus | - | AUACGAGAUGUUGGUGCGGUUCAAUGAGAAAGAAGUUGUUGAAUAAGUUUUUGACCCUACUUUUUGAUUGAGCCGUGCCAAUAUCACGUGU | | | | | |
|  |  |  |  | (((((.((((((((..(((((((((.((((((.((..((..((......))..)).)).)))))).)))))))))..)))))))).))))) | | | | | |
| sly-miR171a |  |  | 418^c^ | ................................................................UGAUUGAGCCGUGCCAAUAUC...... | | | | | |
| sly-miR171a* |  |  | 2 | ........UGUUGGUGCGGUUCAAUGAGA.............................................................. | | | | | |
| sly-miR171b |  |  | 122^e^ | ...................................................................UUGAGCCGUGCCAAUAUCACG... | | | | | |
| sly-miR171b* |  |  | 109 | .....AGAUGUUGGUGCGGUUCAAUG................................................................. | | | | | |
|  |  |  |  |  | | | | | |
| pre-miR171b | SL2.50ch02:44783517..44783603 | minus | - | ACGAGAUAUUGGUGCGGUUCAAUUAGAAAGCGCACUUCUUUAUAUAUAGAACUUCGUUAUUUAAUUGAGCCGUGCCAAUAUCACGUA | | | | | |
|  |  |  |  | (((.((((((((..(((((((((((((.((((...((((........))))...)))).)))))))))))))..)))))))).))). | | | | | |
| sly-miR171b |  |  | 122^e^ | ................................................................UUGAGCCGUGCCAAUAUCACG.. | | | | | |
| sly-miR171b* |  |  | 13 | ...AGAUAUUGGUGCGGUUCAAUU............................................................... | | | | | |
| iso-sly-miR171b |  |  | 1 | ..............................................................AAUUGAGCCGUGCCAAUAUCA.... | | | | | |
| iso-sly-miR171b* |  |  | 13 | .....AUAUUGGUGCGGUUCAAUUAG............................................................. | | | | | |
|  |  |  |  |  | | | | | |
| pre-miR171d | SL2.50ch12:519795..519887 | minus | - | UACACGAGAUAUUGGUGCGGUUCAAUGAGAAAGCAGUACUUAAGAACAUUUUUGAGUCUACUUUUUGAUUGAGCCGCGCCAAUAUCACGUGUA | | | | | |
|  |  |  |  | ((((((.(((((((((((((((((((.((((((.((.((((((((....)))))))))).)))))).))))))))))))))))))).)))))) | | | | | |
| sly-miR171d |  |  | 50 | ....................................................................UUGAGCCGCGCCAAUAUCACG.... | | | | | |
| sly-miR171d* |  |  | 23 | ......AGAUAUUGGUGCGGUUCAAUG.................................................................. | | | | | |
| iso-sly-miR171d |  |  | 23 | .................................................................UGAUUGAGCCGCGCCAAUAUC.... | | | | | |
| iso-sly-miR171d* |  |  | 19 | .........UAUUGGUGCGGUUCAAUGAGA............................................................... | | | | | |
|  |  |  |  |  | | | | | |
| pre-miR171e | SL2.50ch07:60765650..60765756 | plus | - | UAGGAAGAUAUAUAUAGAUAUUGAUGCGGUUCAAUCUGAAAGACAUGGUUAGAUAUGUAAUUAGCCUUGUAAUUUUGGAUUGAGCCGCGUCAAUAUCUCUCUUCCUA | | | | | |
|  |  |  |  | ((((((((.......(((((((((((((((((((((..(((.(((.((((((........)))))).)))..)))..))))))))))))))))))))).)))))))) | | | | | |
| sly-miR171e |  |  | 130 | ...............................................................................UUGAGCCGCGUCAAUAUCUCU....... | | | | | |
| sly-miR171e* |  |  | 66 | ...............AGAUAUUGAUGCGGUUCAAUC....................................................................... | | | | | |
|  |  |  |  |  | | | | | |
| pre-miR171f | SL2.50ch02:33343981..33344081 | minus | - | UGAAAUAGUAACUCUGAUAUUGGCCUGGUUCACUCAGACACAUAUUAUUUUGAUUGAUUUUACUUUUGAUUGAGCCGUGUCAAUAUCUCAGUUCCUUUUCA | | | | | |
|  |  |  |  | .((((.((.((((..(((((((((.(((((((.(((((.....((((.......))))......))))).))))))).)))))))))..)))).)))))). | | | | | |
| sly-miR171f |  |  | 14 | ..................................................................UGAUUGAGCCGUGUCAAUAUC.............. | | | | | |
| sly-miR171f* |  |  | 287^d^ | .................UAUUGGCCUGGUUCACUCAGA............................................................... | | | | | |
|  |  |  |  |  | | | | | |
| pre-miR171g | SL2.50ch07:2683169..2683298 | plus | - | AGUAUAUAUUGAAUUAGAGAAGCGAUGUUGGUGAGGUUCAAUCUGAAGACGAGUUUACGUUUUAUUUCAGUAAAGAACGAUCUCAGAUUGAGCCGCGCCAAUAUCAUUUCUUUUUACCAAUUCAUUUACU | | | | | |
|  |  |  |  | ((((.....(((((((((((((.((((((((((.((((((((((((.((((..(((((...........)))))...)).)))))))))))))).)))))))))).))))))).....))))))..)))) | | | | | |
| sly-miR171g |  |  | 2 | .......................................................................................UUGAGCCGCGCCAAUAUCAUU...................... | | | | | |
| sly-miR171g* |  |  | 4^f^ | ......................CGAUGUUGGUGAGGUUCAAUC....................................................................................... | | | | | |
|  |  |  |  |  | | | | | |
| pre-miR171h | SL2.50ch12:2882790..2882901 | plus | - | AUGGAAGAAGCGAUGUUGGUGAGGUUCAAUCCGAAGACGAAUUUAUGCUUAUUUUCGUAAAGAACGAUCUCAGAUUGAGCCGCGCCAAUAUCACUUCUUAUUUUCAUUCCGU | | | | | |
|  |  |  |  | ((((((((((.((((((((((.(((((((((.((.((((..((((((........))))))...)).)))).))))))))).)))))))))).))))).........))))) | | | | | |
| sly-miR171h |  |  | 11 | ..........................................................................UUGAGCCGCGCCAAUAUCACU................. | | | | | |
| sly-miR171h* |  |  | 4^f^ | ..........CGAUGUUGGUGAGGUUCAAUC................................................................................. | | | | | |
