# Supplementary material for: Functional Characterization of microRNA171 Family in Tomato
Source: Plants (Basel). 2019 Jan 4;8(1):10. doi: 10.3390/plants8010010 (PMC6358981; doi:10.3390/plants8010010)
Supplement: Supplementary file 1 [file plants-08-00010-s001.zip › plants-414116-supplementary-final/Table S2.docx]

**Supplementary Table S2** psRNATarget analysis^a^ of sly-miR171 members and their star strands.

| **miRNA_Acc.** | **Target_Acc.** | **Annotation^b^** | **Expectation** | **UPE$** | **miRNA_start** | **miRNA_end** | **Target_start** | **Target_end** | **miRNA_aligned_fragment** | **Target_aligned_fragment** | **Inhibition** | **Target_Desc.** | | | | |  |  |  |
| --- | --- | --- | --- | --- | --- | --- | --- | --- | --- | --- | --- | --- | --- | --- | --- | --- | --- | --- | --- |
| iso-sly-miR171d | Solyc01g090950 | SlHAM2 | 0 | -1 | 1 | 21 | 972 | 992 | UGAUUGAGCCGCGCCAAUAUC | GAUAUUGGCGCGGCUCAAUCA | Cleavage | Transcription factor GRAS (AHRD V3.3 *** A0A103XI66_CYNCS) | | | | |  |  |  |
| iso-sly-miR171d | Solyc08g078800 | SlHAM | 0 | -1 | 1 | 21 | 1352 | 1372 | UGAUUGAGCCGCGCCAAUAUC | GAUAUUGGCGCGGCUCAAUCA | Cleavage | GRAS family transcription factor (AHRD V3.3 *** A0A061GBD8_THECC) | | | | |  |  |  |
| sly-miR171d | Solyc08g078800 | SlHAM | 0 | -1 | 1 | 21 | 1349 | 1369 | UUGAGCCGCGCCAAUAUCACG | AAUGAUAUUGGCGCGGCUCAA | Cleavage | GRAS family transcription factor (AHRD V3.3 *** A0A061GBD8_THECC) | | | | |  |  |  |
| sly-miR171g | Solyc08g078800 | SlHAM | 0 | -1 | 1 | 21 | 1349 | 1369 | UUGAGCCGCGCCAAUAUCAUU | AAUGAUAUUGGCGCGGCUCAA | Cleavage | GRAS family transcription factor (AHRD V3.3 *** A0A061GBD8_THECC) | | | | |  |  |  |
| sly-miR171h | Solyc08g078800 | SlHAM | 0 | -1 | 1 | 21 | 1349 | 1369 | UUGAGCCGCGCCAAUAUCACU | AAUGAUAUUGGCGCGGCUCAA | Cleavage | GRAS family transcription factor (AHRD V3.3 *** A0A061GBD8_THECC) | | | | |  |  |  |
| sly-miR171d | Solyc01g090950 | SlHAM2 | 1 | -1 | 1 | 21 | 969 | 989 | UUGAGCCGCGCCAAUAUCACG | AGGGAUAUUGGCGCGGCUCAA | Cleavage | Transcription factor GRAS (AHRD V3.3 *** A0A103XI66_CYNCS) | | | | |  |  |  |
| sly-miR171g | Solyc01g090950 | SlHAM2 | 1 | -1 | 1 | 21 | 969 | 989 | UUGAGCCGCGCCAAUAUCAUU | AGGGAUAUUGGCGCGGCUCAA | Cleavage | Transcription factor GRAS (AHRD V3.3 *** A0A103XI66_CYNCS) | | | | |  |  |  |
| sly-miR171h | Solyc01g090950 | SlHAM2 | 1 | -1 | 1 | 21 | 969 | 989 | UUGAGCCGCGCCAAUAUCACU | AGGGAUAUUGGCGCGGCUCAA | Cleavage | Transcription factor GRAS (AHRD V3.3 *** A0A103XI66_CYNCS) | | | | |  |  |  |
| iso-sly-miR171a.1 | Solyc08g078800 | SlHAM | 1.5 | -1 | 1 | 21 | 1348 | 1368 | UGAGCCGUGCCAAUAUCAUCU | AAAUGAUAUUGGCGCGGCUCA | Cleavage | GRAS family transcription factor (AHRD V3.3 *** A0A061GBD8_THECC) | | | | |  |  |  |
| iso-sly-miR171a.2 | Solyc08g078800 | SlHAM | 1.5 | -1 | 1 | 21 | 1349 | 1369 | UUGAGCCGUGCCAAUAUCAUA | AAUGAUAUUGGCGCGGCUCAA | Cleavage | GRAS family transcription factor (AHRD V3.3 *** A0A061GBD8_THECC) | | | | |  |  |  |
| sly-miR171a | Solyc01g090950 | SlHAM2 | 1.5 | -1 | 1 | 21 | 972 | 992 | UGAUUGAGCCGUGCCAAUAUC | GAUAUUGGCGCGGCUCAAUCA | Cleavage | Transcription factor GRAS (AHRD V3.3 *** A0A103XI66_CYNCS) | | | | |  |  |  |
| sly-miR171a | Solyc08g078800 | SlHAM | 1.5 | -1 | 1 | 21 | 1352 | 1372 | UGAUUGAGCCGUGCCAAUAUC | GAUAUUGGCGCGGCUCAAUCA | Cleavage | GRAS family transcription factor (AHRD V3.3 *** A0A061GBD8_THECC) | | | | |  |  |  |
| sly-miR171b | Solyc08g078800 | SlHAM | 1.5 | -1 | 1 | 21 | 1349 | 1369 | UUGAGCCGUGCCAAUAUCACG | AAUGAUAUUGGCGCGGCUCAA | Cleavage | GRAS family transcription factor (AHRD V3.3 *** A0A061GBD8_THECC) | | | | |  |  |  |
| iso-sly-miR171a.2 | Solyc01g090950 | SlHAM2 | 2.5 | -1 | 1 | 21 | 969 | 989 | UUGAGCCGUGCCAAUAUCAUA | AGGGAUAUUGGCGCGGCUCAA | Cleavage | Transcription factor GRAS (AHRD V3.3 *** A0A103XI66_CYNCS) | | | | |  |  |  |
| iso-sly-miR171a.2 | Solyc02g085600 | SlHAM4 | 2.5 | -1 | 1 | 21 | 582 | 602 | UUGAGCCGUGCCAAUAUCAUA | UGUGAUACUGGCACGCCUCAA | Cleavage | Transcription factor GRAS (AHRD V3.3 *** A0A103YGV0_CYNCS) | | | | |  |  |  |
| iso-sly-miR171b | Solyc08g078800 | SlHAM | 2.5 | -1 | 1 | 21 | 1351 | 1371 | AAUUGAGCCGUGCCAAUAUCA | UGAUAUUGGCGCGGCUCAAUC | Translation | GRAS family transcription factor (AHRD V3.3 *** A0A061GBD8_THECC) | | | | |  |  |  |
| iso-sly-miR171b | Solyc01g090950 | SlHAM2 | 2.5 | -1 | 1 | 21 | 971 | 991 | AAUUGAGCCGUGCCAAUAUCA | GGAUAUUGGCGCGGCUCAAUC | Translation | Transcription factor GRAS (AHRD V3.3 *** A0A103XI66_CYNCS) | | | | |  |  |  |
| iso-sly-miR171b | Solyc02g086540 | NA | 2.5 | -1 | 1 | 21 | 4560 | 4580 | AAUUGAGCCGUGCCAAUAUCA | AAAAAUUGGCACAGCUCAAUU | Cleavage | Core-2/I-branching beta-1,6-N-acetylglucosaminyltransferase family protein (AHRD V3.3 *** AT5G14550.1) | | | | |  |  |  |
| sly-miR171a | Solyc02g085600 | SlHAM4 | 2.5 | -1 | 1 | 21 | 585 | 605 | UGAUUGAGCCGUGCCAAUAUC | GAUACUGGCACGCCUCAAUCA | Cleavage | Transcription factor GRAS (AHRD V3.3 *** A0A103YGV0_CYNCS) | | | | |  |  |  |
| sly-miR171b | Solyc01g090950 | SlHAM2 | 2.5 | -1 | 1 | 21 | 969 | 989 | UUGAGCCGUGCCAAUAUCACG | AGGGAUAUUGGCGCGGCUCAA | Cleavage | Transcription factor GRAS (AHRD V3.3 *** A0A103XI66_CYNCS) | | | | |  |  |  |
| sly-miR171b | Solyc02g085600 | SlHAM4 | 2.5 | -1 | 1 | 21 | 582 | 602 | UUGAGCCGUGCCAAUAUCACG | UGUGAUACUGGCACGCCUCAA | Cleavage | Transcription factor GRAS (AHRD V3.3 *** A0A103YGV0_CYNCS) | | | | |  |  |  |
| sly-miR171e | Solyc01g090950 | SlHAM2 | 2.5 | -1 | 1 | 21 | 969 | 989 | UUGAGCCGCGUCAAUAUCUCU | AGGGAUAUUGGCGCGGCUCAA | Translation | Transcription factor GRAS (AHRD V3.3 *** A0A103XI66_CYNCS) | | | | |  |  |  |
| sly-miR171e | Solyc08g078800 | SlHAM | 2.5 | -1 | 1 | 21 | 1349 | 1369 | UUGAGCCGCGUCAAUAUCUCU | AAUGAUAUUGGCGCGGCUCAA | Translation | GRAS family transcription factor (AHRD V3.3 *** A0A061GBD8_THECC) | | | | |  |  |  |
| sly-miR171f | Solyc01g090950 | SlHAM2 | 2.5 | -1 | 1 | 21 | 972 | 992 | UGAUUGAGCCGUGUCAAUAUC | GAUAUUGGCGCGGCUCAAUCA | Cleavage | Transcription factor GRAS (AHRD V3.3 *** A0A103XI66_CYNCS) | | | | |  |  |  |
| sly-miR171f | Solyc08g078800 | SlHAM | 2.5 | -1 | 1 | 21 | 1352 | 1372 | UGAUUGAGCCGUGUCAAUAUC | GAUAUUGGCGCGGCUCAAUCA | Cleavage | GRAS family transcription factor (AHRD V3.3 *** A0A061GBD8_THECC) | | | | |  |  |  |
| iso-sly-miR171a.1* | Solyc06g076250 |  | 2.5 | -1 | 1 | 21 | 951 | 971 | AUGAUGUUGGAAUGGCUCAAU | GUUGAGACAUGCCAACAUCAU | Translation | PGR5-like protein 1B, chloroplastic (AHRD V3.3 *** A0A0B0MIW0_GOSAR) | | | | |  |  |  |
| iso-sly-miR171a.1* | Solyc02g089263 |  | 2.5 | -1 | 1 | 21 | 10044 | 10064 | AUGAUGUUGGAAUGGCUCAAU | GGUCAGCCAUUUCAACAUCAU | Translation | auxin transport protein (BIG) (AHRD V3.3 *** AT3G02260.3) | | | | |  |  |  |
| sly-miR171e* | Solyc01g110220 |  | 3 | -1 | 1 | 21 | 177 | 197 | AGAUAUUGAUGCGGUUCAAUC | GCUUCCACCGCAUCAAUAUCC | Cleavage | LOW QUALITY:MEI2 C-terminal RRM only like 2 (AHRD V3.3 *-* AT5G07930.3) | | | | |  |  |  |
|  |  |  |  |  |  |  |  |  |  |  |  |  | | | | |  |  |  |
|  | | | | | | | | | | | | | |  | |  | |  |  |
|  | | | | | | | | | | | | | | | | | | |  |
| ^a^Anlysis was done against cDNA library ITAG 3.1 using default parametes of Schema V2 (2017 release) | | | | | | | | | | | | | |  | |  | |  | |
| ^b^ Hendelman, A., Kravchik, M., Stav, R., Frank, W. and Arazi, T. (2016) Tomato HAIRY MERISTEM genes are involved in meristem maintenance and compound leaf morphogenesis. Journal of Experimental Botany, 67, 6187–6200. | | | | | | | | | | | | | | | | | | | |
