# Supplementary material for: Functional Characterization of microRNA171 Family in Tomato
Source: Plants (Basel). 2019 Jan 4;8(1):10. doi: 10.3390/plants8010010 (PMC6358981; doi:10.3390/plants8010010)
Supplement: Supplementary file 1 [file plants-08-00010-s001.zip › plants-414116-supplementary-final/Table S3.docx]

| Table S3. Primers and probes used in this study | |  |  |
| --- | --- | --- | --- |
| **Primer ID** | **Primer sequence (5' – 3')^abc^** | **Gene ID** | **Remarks** |
| sly-miR171a_RC | GATATTGGCACGGCTCAATCA |  | miR probes |
| sly-miR171b_RC | CGTGATATTGGCACGGCTCAA |  |  |
| sly-miR171e_RC | AGAGATATTGACGCGGCTCAA |  |  |
| sly-miR159_RC | TAGAGCTCCCTTCAATCCAAA |  |  |
| U6_RC | AGGGGCCATGCTAATCTTCTC |  | U6 snRNA probe |
| OCS_rev | GAAACCGGCGGTAAGGATCT |  | Transgene validatin |
| Xho_STTM_171_F | CCGCTCGAGGATATTGGCGTAAAGGCTCAATCAGTTGTTGTT |  | Plasmid construction |
| Hind_STTM_171_R | CCCAAGCTTTTGAGCCGCGTTTCCAATATCACTATTCTTCTTCT |  |  |
| Xho_MIR171e_F | CCGCTCGAGTGAAGTTGGGAAATATTGAAGG |  |  |
| Hind_MIR171e_R | CCCAAGCTTGGCCAAGAGGAAAACATGAA |  |  |
| qRT-SGN-U569651_F | TGTTGGTGTTGGCATGGAGTAGCA | *Solyc01g090950* | Real time primers |
| qRT-SGN-U569651_R | GCAGAGCTGGTCCAGACAGGGA |  |  |
| qRT-SGN-U592620_F | TTCAGGCCTCTGAACTATTGCT | *Solyc08g078800* |  |
| qRT-SGN-U592620_R | CAACTGCAGAGCCTCCTTGATA |  |  |
| qRT-SGN-U600069_F | GCGGAGGCATTAACCGGCGT | *Solyc11g013150* |  |
| qRT-SGN-U600069_R | TGTAACGTACCGGAAGCCATCA |  |  |
| qRT-TIP41_F | ATGGAGTTTTTGAGTCTTCTGC | *Solyc10g049850* |  |
| qRT-TIP41_R | GCTGCGTTTCTGGCTTAGG |  |  |
| ^a^Sequences corresponding to restriction enzyme sites are underlined and designated by red. | |  |  |
