# Supplementary figures and images for: Functional Characterization of microRNA171 Family in Tomato
Source: Plants (Basel). 2019 Jan 4;8(1):10. doi: 10.3390/plants8010010 (PMC6358981; doi:10.3390/plants8010010)

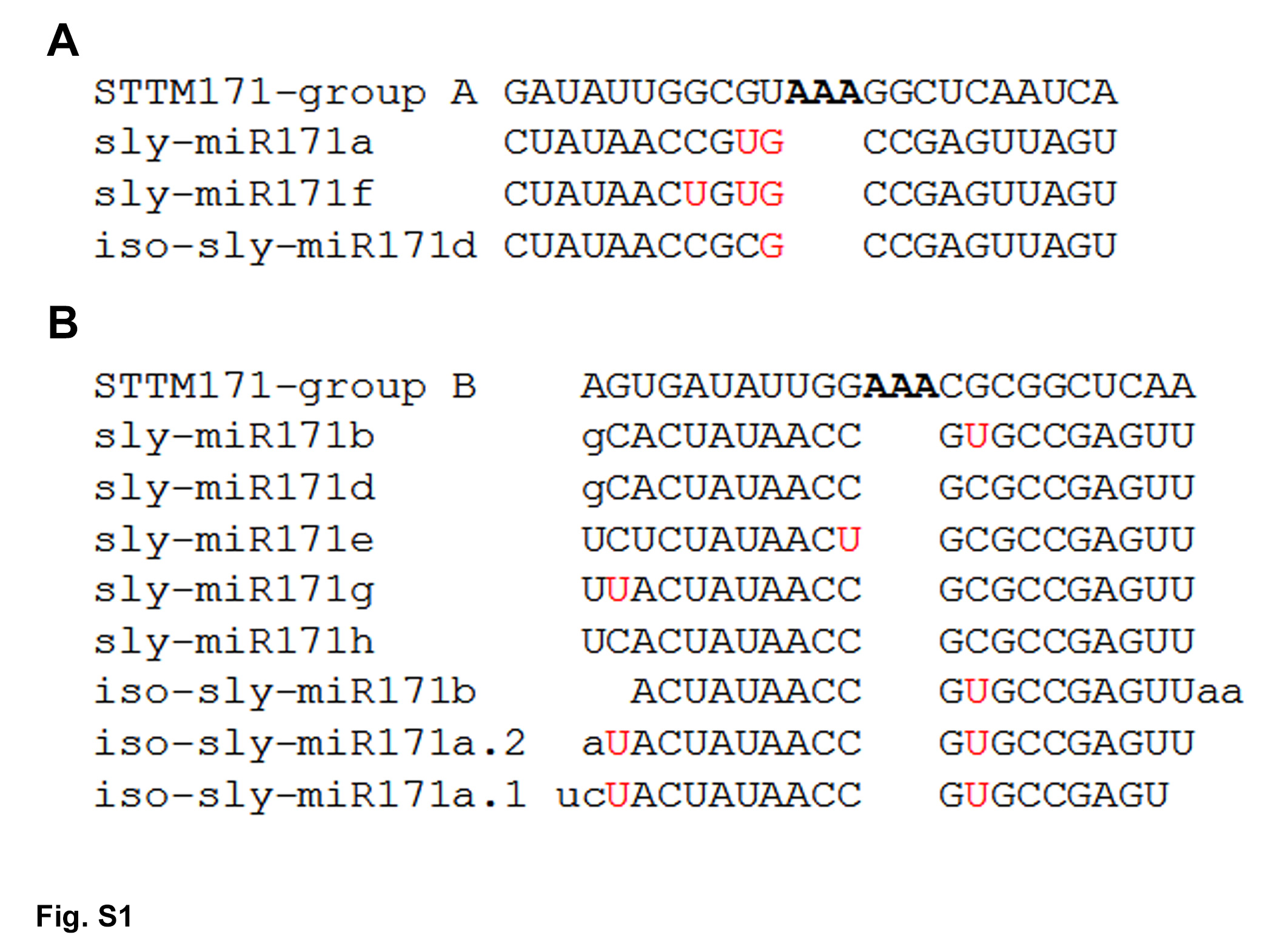

Supplement: Supplementary file 1 [file plants-08-00010-s001.zip › plants-414116-supplementary-final/Figure S1.tif]

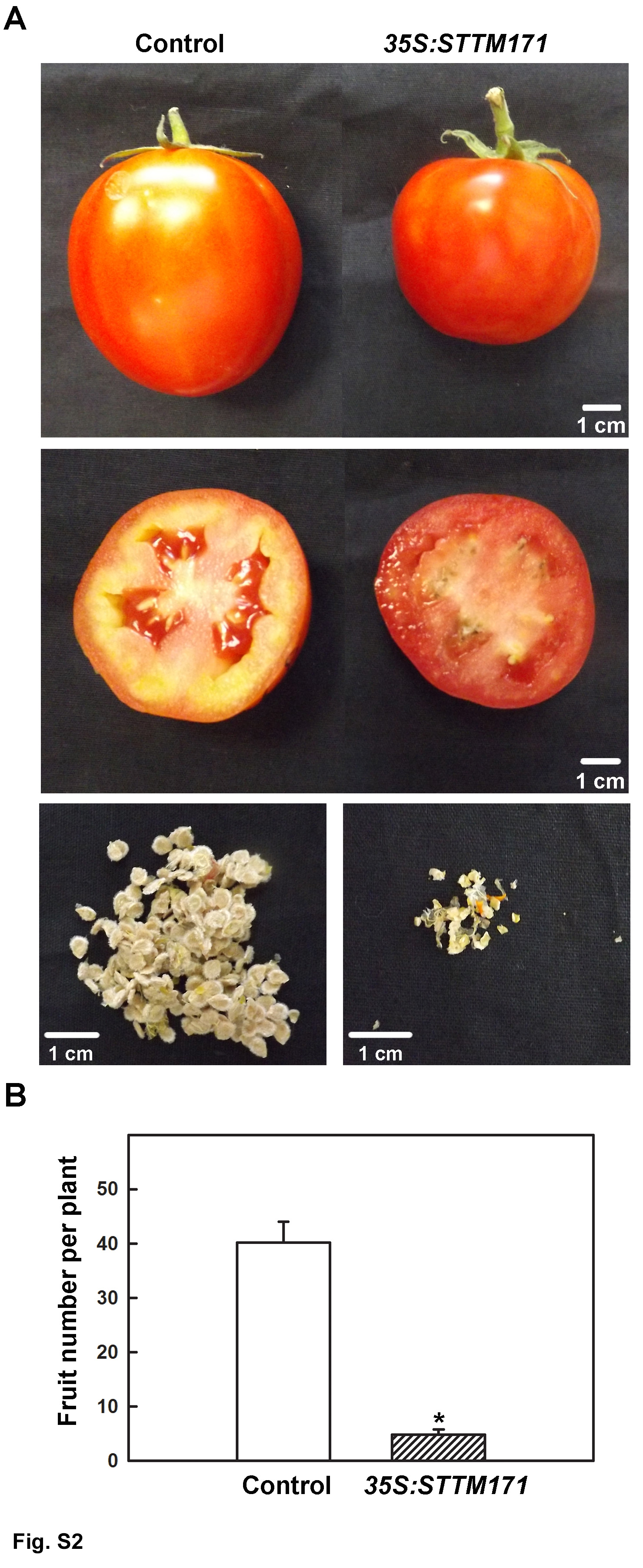

Supplement: Supplementary file 1 [file plants-08-00010-s001.zip › plants-414116-supplementary-final/Figure S2.tif]

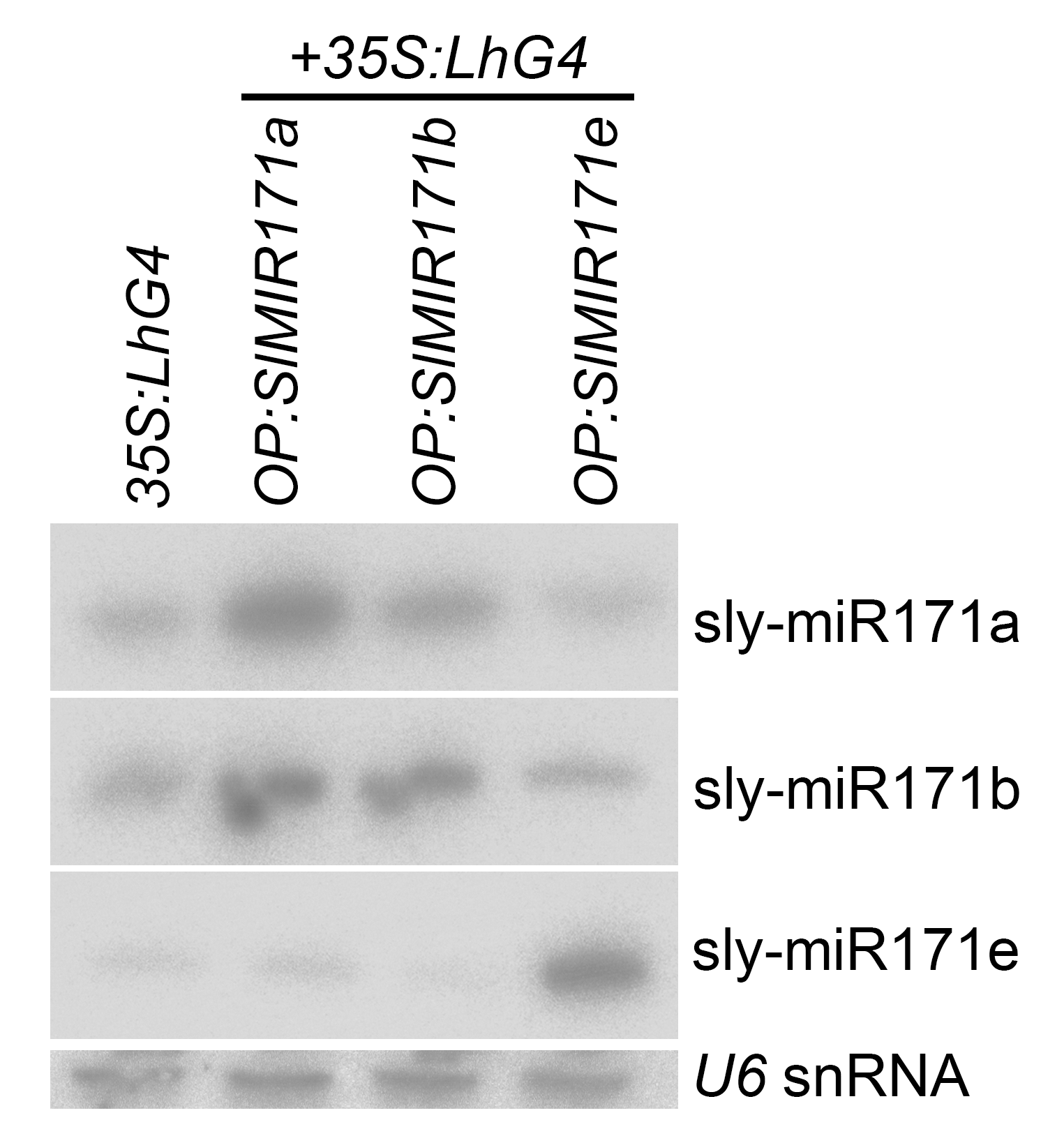

Supplement: Supplementary file 1 [file plants-08-00010-s001.zip › plants-414116-supplementary-final/Figure S3.tif]

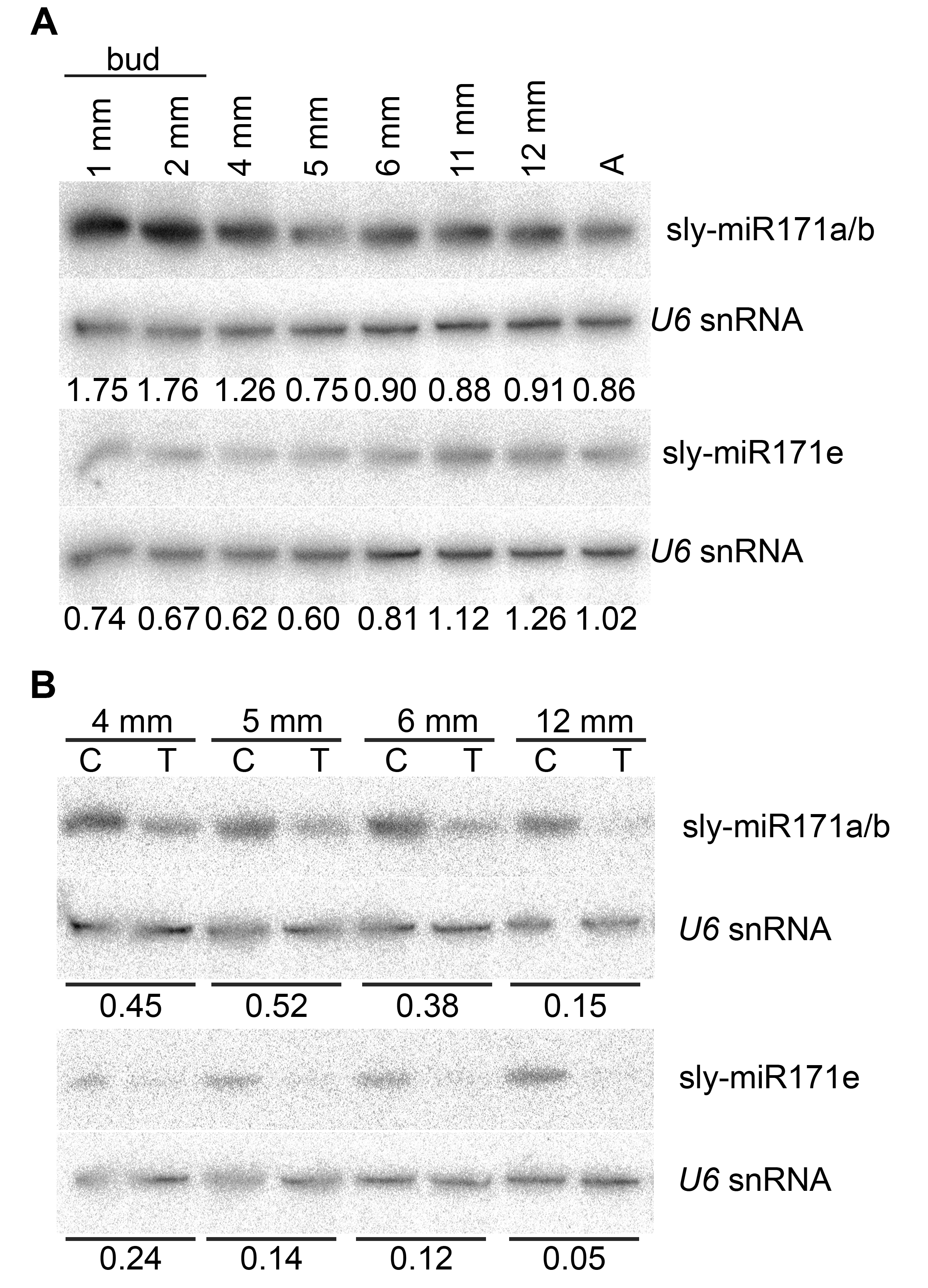

Supplement: Supplementary file 1 [file plants-08-00010-s001.zip › plants-414116-supplementary-final/Figure S4.tif]
